# PHIV-RootCell: a supervised image analysis tool for rice root anatomical parameter quantification

**DOI:** 10.3389/fpls.2014.00790

**Published:** 2015-01-19

**Authors:** Marc Lartaud, Christophe Perin, Brigitte Courtois, Emilie Thomas, Sophia Henry, Mathilde Bettembourg, Fanchon Divol, Nadege Lanau, Florence Artus, Charlotte Bureau, Jean-Luc Verdeil, Gautier Sarah, Emmanuel Guiderdoni, Anne Dievart

**Affiliations:** ^1^CIRAD, UMR AGAPMontpellier, France; ^2^Plateforme Histocytologie et Imagerie Cellulaire Végétale, INRA-CIRADMontpellier, France

**Keywords:** cell number, image analysis software, rice, root, tissue area, transverse histological section, histological phenotype scoring

## Abstract

We developed the PHIV-RootCell software to quantify anatomical traits of rice roots transverse section images. Combined with an efficient root sample processing method for image acquisition, this program permits supervised measurements of areas (those of whole root section, stele, cortex, and central metaxylem vessels), number of cell layers and number of cells per cell layer. The PHIV-RootCell toolset runs under ImageJ, an independent operating system that has a license-free status. To demonstrate the usefulness of PHIV-RootCell, we conducted a genetic diversity study and an analysis of salt stress responses of root anatomical parameters in rice (*Oryza sativa* L.). Using 16 cultivars, we showed that we could discriminate between some of the varieties even at the 6 day-olds stage, and that tropical japonica varieties had larger root sections due to an increase in cell number. We observed, as described previously, that root sections become enlarged under salt stress. However, our results show an increase in cell number in ground tissues (endodermis and cortex) but a decrease in external (peripheral) tissues (sclerenchyma, exodermis, and epidermis). Thus, the PHIV-RootCell program is a user-friendly tool that will be helpful for future genetic and physiological studies that investigate root anatomical trait variations.

## INTRODUCTION

How cells and tissues, notably in roots, are organized and correlated with plant functions is of major interest to understanding plant adaptation to stresses. High throughput phenotypic profiling of root anatomical and architectural traits is critical for quantitative trait loci (QTL) and association mapping (Ron et al., 2013). Studies of anatomical traits notably rely in part on image-based experiments to analyze and extract features from microscopy data.

With advances in microscopy and the automation of sample preparation, it is currently relatively easy to collect 1000s of pictures from large-scale screens. However, the measurement of many features from these images makes analyzing the microscopy data a bottleneck in many experiments. To address this issue, a large number of programs designed specifically for biological image processing and data collection have been developed. They are often highly specialized for specific biological samples, and they are not easily adaptable to different issues and/or are not freely available (Carpenter et al., 2006; Burton et al., 2012; Federici et al., 2012; Pound et al., 2012). The Java-based ImageJ package^1^ offers attractive features such as its license-free status, its operating system independence and its large user community (Schneider et al., 2012). Taking advantage of all the tools already available in ImageJ, we developed a new toolset called PHIV-RootCell.

The PHIV-RootCell program is dedicated to the analysis of several root anatomical parameters based on images of rice root transverse sections. The program tracks root tissues and cell walls to measure areas and numbers of cells in cell files. PHIV-RootCell uses a semi-automated approach where the user has to supervise each step of the process and can proceed to corrections if not satisfied with the software’s proposals. Data are exported as tabulated text files that can be directly used for statistical analyses.

## MATERIALS AND METHODS

### PLANT MATERIAL AND GROWTH CONDITIONS

For all experiments, rice (*Oryza sativa*) seedlings were grown vertically in sterile square petri dishes (Corning, 431301; 20 cm × 20 cm) under controlled conditions (day/night temperature of 28/25°C, a 12 h photoperiod, and a light intensity of 500 μEm^-2^s^-1^). First, the seeds are surface-sterilized: seeds are rinsed in 70% ethanol for ∼1 min. Then, ethanol is replaced by a solution composed of 40% bleach in distilled water containing 0.4% Tween 80 (Sigma-Aldrich P4780-500 mL). The seeds are soaked in this solution for 30 min with gentle agitation, and then rinsed at least four times with sterile distilled water. The sterile seeds are then sown on square petri dishes containing 250 mL of half strength Murashige and Skoog (MS/2) solid medium with the radicle oriented downward. Ten seeds are plated on each petri dish. The MS/2 solid medium is composed of 2.15 g/L of Murashige and Skoog medium basal salt mixture (Duchefa Biochemie, M0221), 75 mg/L Murashige and Skoog vitamin mixture (Duchefa Biochemie, M0409) and 8 g/L of agarose type II (Sigma-Aldrich, A6877). For salt-stress experiments, 7 g/L of NaCl (120 mM) is added to MS/2 medium before autoclaving. Radicles of the plantlets were harvested after 6 days of growth.

### AGAROSE ROOT EMBEDDING AND SECTIONING

Root tips (∼2 cm long) of growing radicles are cut with a sharp blade. Five different root tip explants are placed parallel to one another to make the root tips aligned and embedded in a drop of hot (∼60°C) liquid 3% agarose in water. When the agarose is solidified, this patch containing the roots is inserted vertically in a well-filled with hot liquid 3% agarose. After solidification, the blocks are resized and glued on a vibratome plate to make sections 60 μm in thickness at 0.5 cm from the root tip with an Hm650v vibratome (Thermo Scientific Microm, speed 30–50, frequency 70, amplitude 0.8). Individual sections are then collected with a fine brush, transferred to slides (humidified with 1X phosphate buffered saline (PBS, Sigma-Aldrich P3813) and covered with a coverslip for direct observation.

### IMAGE ACQUISITION AND PROCESSING

To assess transverse section parameters using autofluorescence of cell walls, pictures were taken with a DM6000 B epifluorescence microscope (Leica) equipped with an ‘A’ filter cube (excitation range: UV; excitation filter: BP 340–380; suppression filter: LP 425). Images were taken using a color Retiga 2000R camera (QIMAGING, Canada) running Volocity image acquisition software (Improvision, UK). When the program is launched, the RGB image is transformed in a gray level (8-bit) image, then a Gaussian filter is applied. The contrast is automatically enhanced and the threshold can be manually adjusted at each step of the analysis to create the selection. Then the selection is enlarged and decreased to smooth it. For cell count, a polar transformation is applied. This allows to draw a line on which local maxima will be counted. The noise tolerance parameter can be adjusted depending on image quality.

## RESULTS

### A HIGH THROUGHPUT PROTOCOL FOR SECTIONING RADICLE ROOTS OF RICE PLANTLETS

**FIGURE 1:**
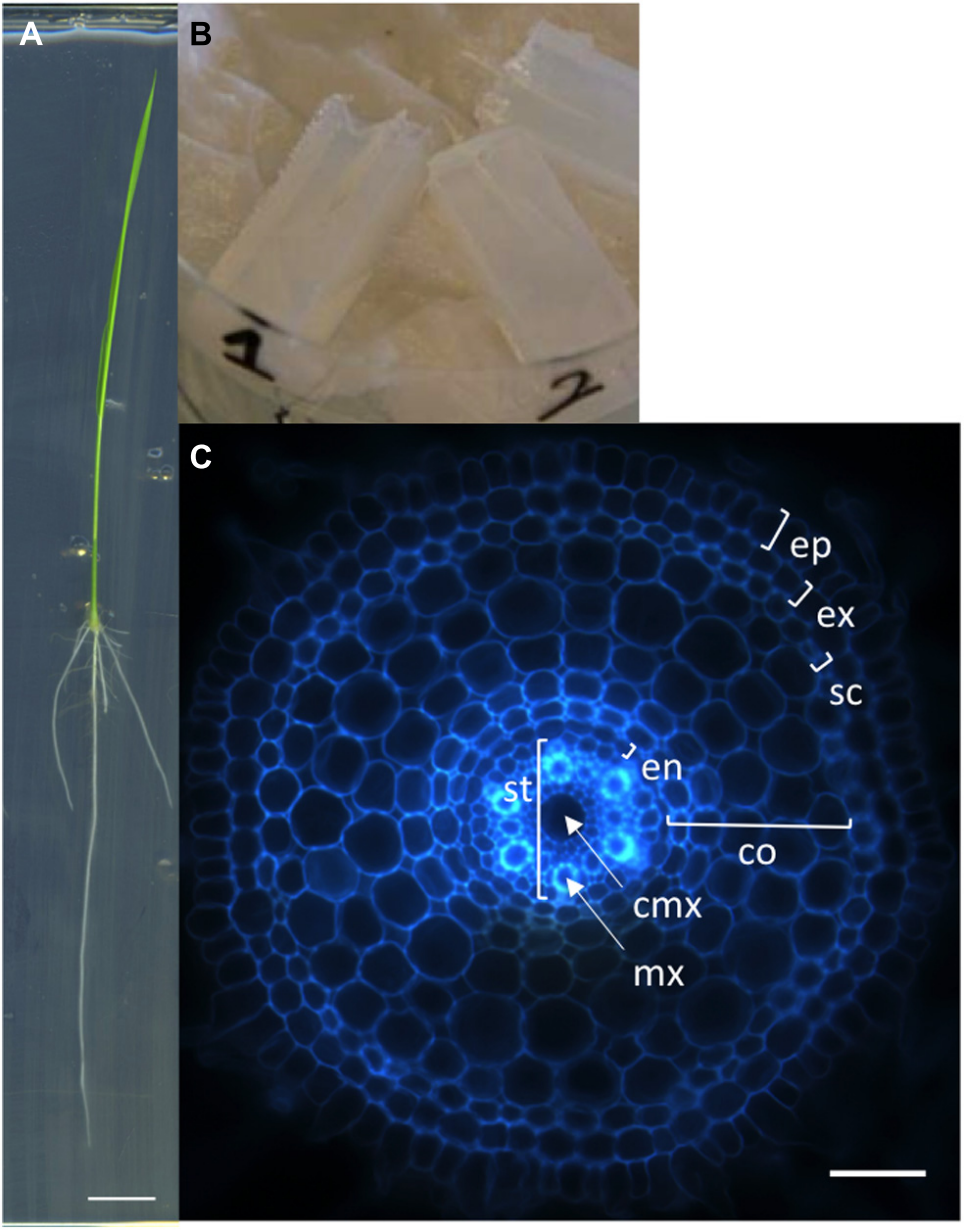
**Nipponbare seedling, agarose blocks, and radicle transverse section. (A)** A rice seedling grown vertically on MS/2 medium for 6 days. **(B)** Picture of agarose blocks containing several root tips. These blocks are ready to be resized for vibratome sectioning. **(C)** Autofluorescence image of a transverse histological root section. The rice root tissues are concentric cylinders; from external to internal, the cell layers are the epidermis (ep), exodermis (ex), sclerenchyma (sc), several layers of cortex (co), endodermis (en), and pericycle. The pericycle delimits the stele (st) containing the vascular vessels [the central metaxylem (cmx) and the metaxylems (mx)]. Scale bars: A 1 cm, C 100 μm.

We first developed a simple, rapid and robust protocol for producing transverse sections of rice radicle meristems from fresh tissues. Sterile rice seeds are sown on square petri dishes containing solid MS/2 medium and the plantlets are grown vertically for 6 days (**Figure 1A**). The root tips are then cut with a sharp blade and embedded into agarose blocks for sectioning with a vibratome (**Figure 1B**). Each transverse section is observed and photographed under an epifluorescent microscope taking advantage of the natural autofluorescence of plant root cell walls (**Figure 1C**). Using this protocol, one person can easily produce cross-sections of more than 100 root samples in 1 day. This protocol has allowed us to generate a large number of pictures. The manual measurement of anatomical root traits from these pictures was highly tedious and time-consuming. Therefore, we designed an efficient tool to semi-automatically analyze these images.

### SPECIFICATIONS, REQUIREMENTS, AND INSTALLATION OF THE PHIV-ROOTCELL TOOLSET

The PHIV-RootCell program is a macro toolset running on the 1.48 h (or higher) version of ImageJ^2^. The “Polar Transformer” plugin is needed^3^ and has to be saved in the plugins folder (ImageJ/plugins/). All the files related to PHIV-RootCell [user manual, toolset (text file) and examples] are provided as supplementary files. The text file entitled “PHIV_Rootcell_toolset.txt” has to be saved in the ImageJ/macros/toolsets folder. When starting ImageJ, the plugin becomes accessible in the “More Tools” menu (⟨⟨>>⟩⟩). When the “PHIV_Rootcell_toolset” is selected, nine new tool buttons appear in the toolbar. The name of each tool is displayed in the status bar when the mouse is moved over it [Parameters, Root Selection (R), Stele Selection (S), Xylem Selection and Count (X), Cortex Selection (C), Layer and Cell File Counts (L), Data Display (D), New Image (N), and About These Macros (?)].

### DESCRIPTION OF THE PHIV-ROOTCELL TOOLSET

**FIGURE 2:**
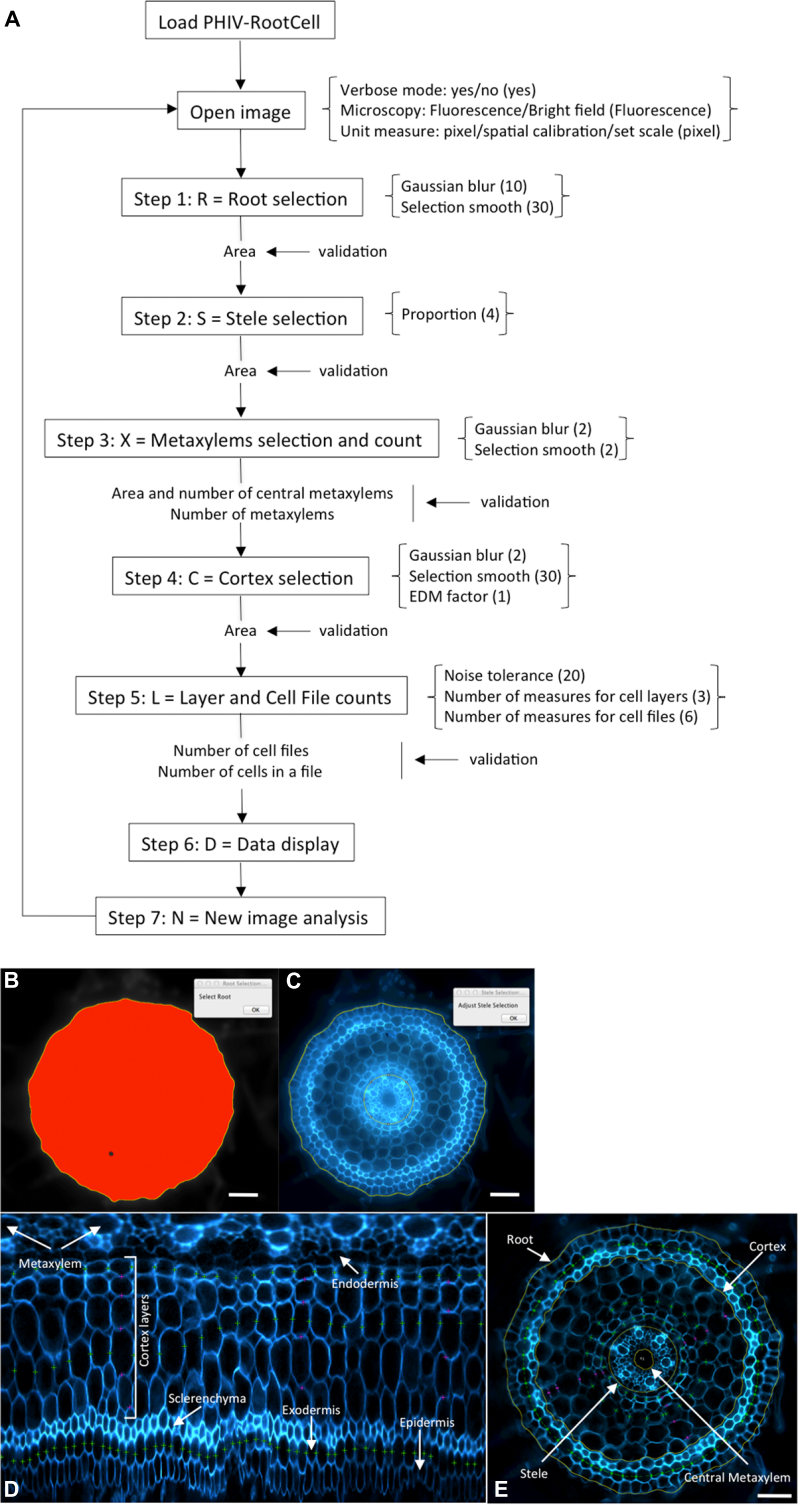
**Workflow and screen captures from the PHIV-RootCell toolset. (A)** Workflow of the PHIV-RootCell program. All the steps are described schematically with parameters associated under brackets. Default parameters are under parenthesis. **(B–E)** Examples of automatic selections of the entire root **(B)**, of the stele **(C)** and of a polar transformed image **(D)**. The center for the polar transformation is given by the stele selection. In this way, the metaxylem vessels are at the top of the image and the epidermis at the bottom. **(E)** All parameters analyzed with the PHIV-RootCell toolset replaced on the original picture. Yellow round lines: root, cortex, stele, and central metaxylem edges. The area of the cortex is defined as the measured cortex area minus the stele area. The external (peripheral) layer area (epidermis, exodermis, and sclerenchyma) is calculated by subtracting the measured cortex area from the root area. Magenta spots: three measurements of the number of cortex layers yield the mean number of cortex layers. Green spots: on this image, three cell files have been counted (the exodermis and two cortex files). These magenta and green spots are defined on the polar transformed image **(D)**. Scale bars: 100 μm.

The power of the PHIV-RootCell toolset relies on the fact that the user can correct the automatic detection and supervise each step of the analysis (**Figure 2A**). Three options can be selected using the first optional tool named Parameters: (i) Verbose mode, to be guided or not while running the toolset (default = yes), (ii) Microscopy: fluorescence or bright-field pictures (default = fluorescence), and (iii) Unit measure: pixel, image spatial calibration, or scale set for each image (default = pixel). To analyze root cross-sections, the next seven tools have to be run sequentially. All data will be stored as *Region of Interest* (ROI) in ImageJ. The R button (Root Selection) will automatically select the entire root area (**Figure 2B**). The S button (Stele Selection) will draw an oval selection for the stele area (**Figure 2C**). The size of the oval selection is a parameter of this tool defined as the Root Area/Stele Area proportion (right click, default = 4). The X button (Xylem Selection and Count) will automatically select the central metaxylem area and count the number of vessels. The user will also be asked to count the number of metaxylem vessels and confirm the software’s proposal. The C button (Cortex Selection) will select the tissue area internal to the sclerenchyma. The ⟨⟨External Layer Area⟩⟩, defined as the “root” area minus the “cortex” area, will be calculated. The L button (Layer and Cell File Counts) will first do a polar transformation of the original image (**Figure 2D**). Cell layer number (average of *n* measures, *n* defined by the user as an option, default = 3) and cell number in a particular cell file (the number of cell files to be analyzed is defined by the user as an option, default = 6) are defined by the number of maxima detected in vertical and horizontal lines, respectively, in the polar transformed image. Again, the user will be asked to check and confirm the software’s proposals. For the horizontal lines, if the image quality is not consistent for the entire distance, the user can trace a line on a limited portion of the image. The toolset will then extrapolate the number of cells in the entire image-based on the portion of the image that has been covered. The D button (Data Display) will display and save the image with superimposed selections and ROIs in the original image directory (**Figure 2E**). All the measurements are also shown in the “Analyses” window (see Table S1 for an example of an output table). The N button (New image) will close all images, reset the ROI manager and open the image directory. On average, a trained user will spend from 3 to 5 min per image.

### ROBUSTNESS OF THE PHIV-ROOTCELL TOOLSET

To assay the reproducibility of the software and the effect of manual corrections on the results, five users analyzed the same dataset composed of 10 biological replicates of Nipponbare cultivar cross-sections to quantify 11 parameters (Figure S1). Statistical analyses suggest that, for the selected images, there is no significant user effect for any of the traits analyzed except for the number of cells in the exodermis layer. Despite a low coefficient of variation (3.8%) for this parameter, one of the users overestimated it. Because this value can be extrapolated from a portion of the image, a small difference in the real count of cells will be systematically amplified. However, for this parameter, the difference observed between users only varies in average of four cells out of 61. In conclusion, the tool can be considered to be robust and user-independent.

### EVALUATION OF GENETIC DIVERSITY AND ENVIRONMENTAL RESPONSES OF RICE ROOT ANATOMICAL TRAITS

#### Analysis of root parameters of 16 rice varieties

**FIGURE 3:**
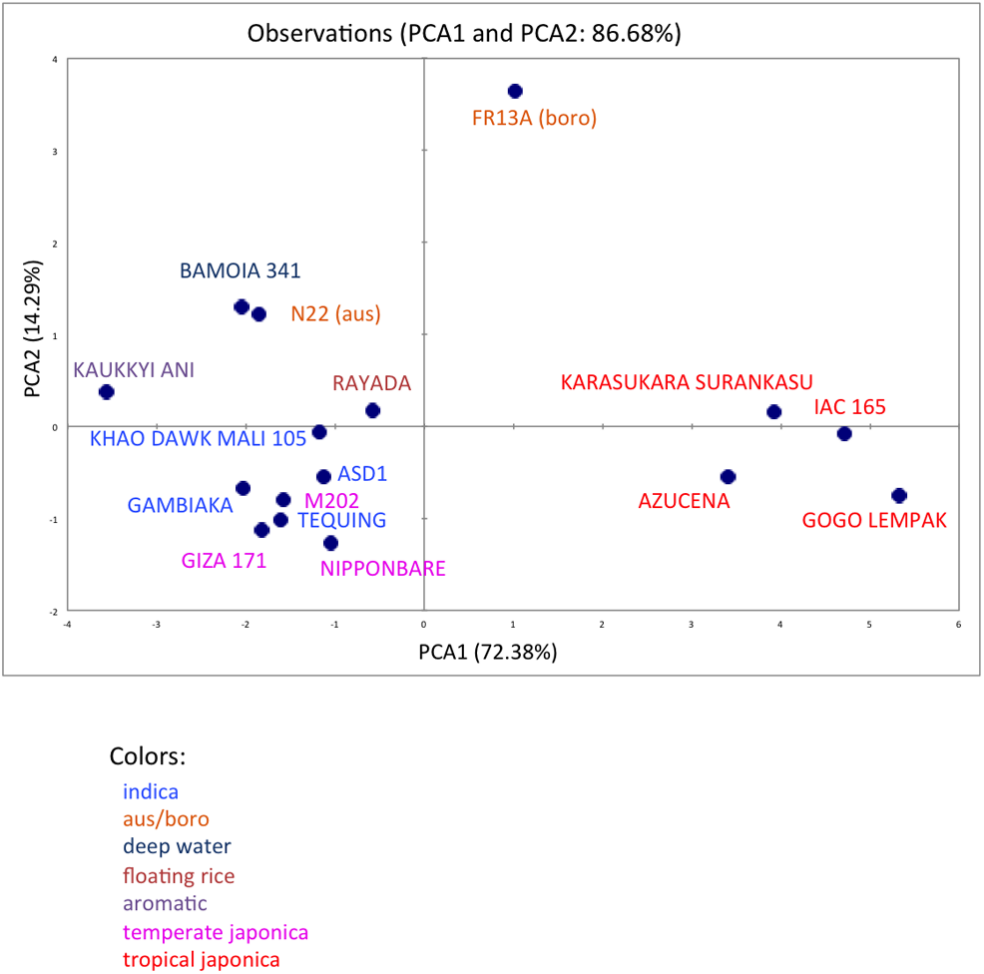
**Principal component analysis of 10 root anatomical parameters measured on 16 varieties of rice.** Projection of the 16 varieties on the two main axes (PCA1 and PCA2). The names of the varieties are indicated, and their colors are based on their membership in specific varietal groups.

We analyzed ten anatomical features on 16 rice varieties belonging to seven different varietal groups (Tables 1 and 2; Glaszmann, 1987). A minimum of four images was analyzed per variety. Significant differences between varieties were recorded for all traits (Figure S2). As expected, there are significant redundancies between areas measured due to allometric growth constraints as seen in the correlation matrix (Table S2). A principal component analysis (PCA) was run on the varietal means for all traits. The two first axes of the PCA explain more than 86% of the variability (**Figure 3**; Figure S3). On the first axis (72% of the data dispersion), two distinct groups are clearly visible. The first group clusters the four tropical japonica varieties with thick roots, and the second group gathers all the other varieties with thinner roots. The second axis is mostly determined by the presence of an outlier, FR13A, belonging to the boro group. All of the rice cultivars analyzed here have fewer than two central metaxylem vessels on average while FR13A has four. Interestingly, the boro variety analyzed (FR13A) is separated from the aus variety (N22) despite their genetic proximity. Due to the small subset of rice varieties analyzed here, it is impossible to say whether this parameter discriminates between the aus and boro accessions, but it is an interesting area to explore. Our data, however, clearly demonstrate that the PHIV-RootCell program can be used to quantify and describe anatomical parameters for genetic analyses of a larger number of varieties, including QTL and genome-wide association studies (GWASs) analyses.

**Table 1:** List of the varieties analyzed.

Varieties	Varietal groups
ASD1	Indica
KHAO DAWK MALI 105	
TEQUING	
GAMBIAKA

FR13A	Boro

N 22	Aus

BAMOIA 341	Deep water

RAYADA	Floating rice

KAUKKYI ANI	Aromatic

IAC 165	Tropical japonica
KARASUKARA SURANKASU	
AZUCENA	
GOGO LEMPAK	

GIZA 171	Temperate japonica
NIPPONBARE	
M 202

**Table 2:** List of the root parameters analyzed.

Parameters analyzed	Abbreviations
Root area	ROOTA
External layer area	ELA
Cortex area	CTXA
Stele area	STELEA
Central metaxylem area	CMA
Number of central metaxylems	NCM
Number of metaxylem vessels	NM
Number of cortex layers	NCL
Number of cells in a cortex layer	NCF1
Number of cells in the exodermis layer	NCF2

#### NaCl treatment increases tissue and cell areas in Nipponbare

**FIGURE 4:**
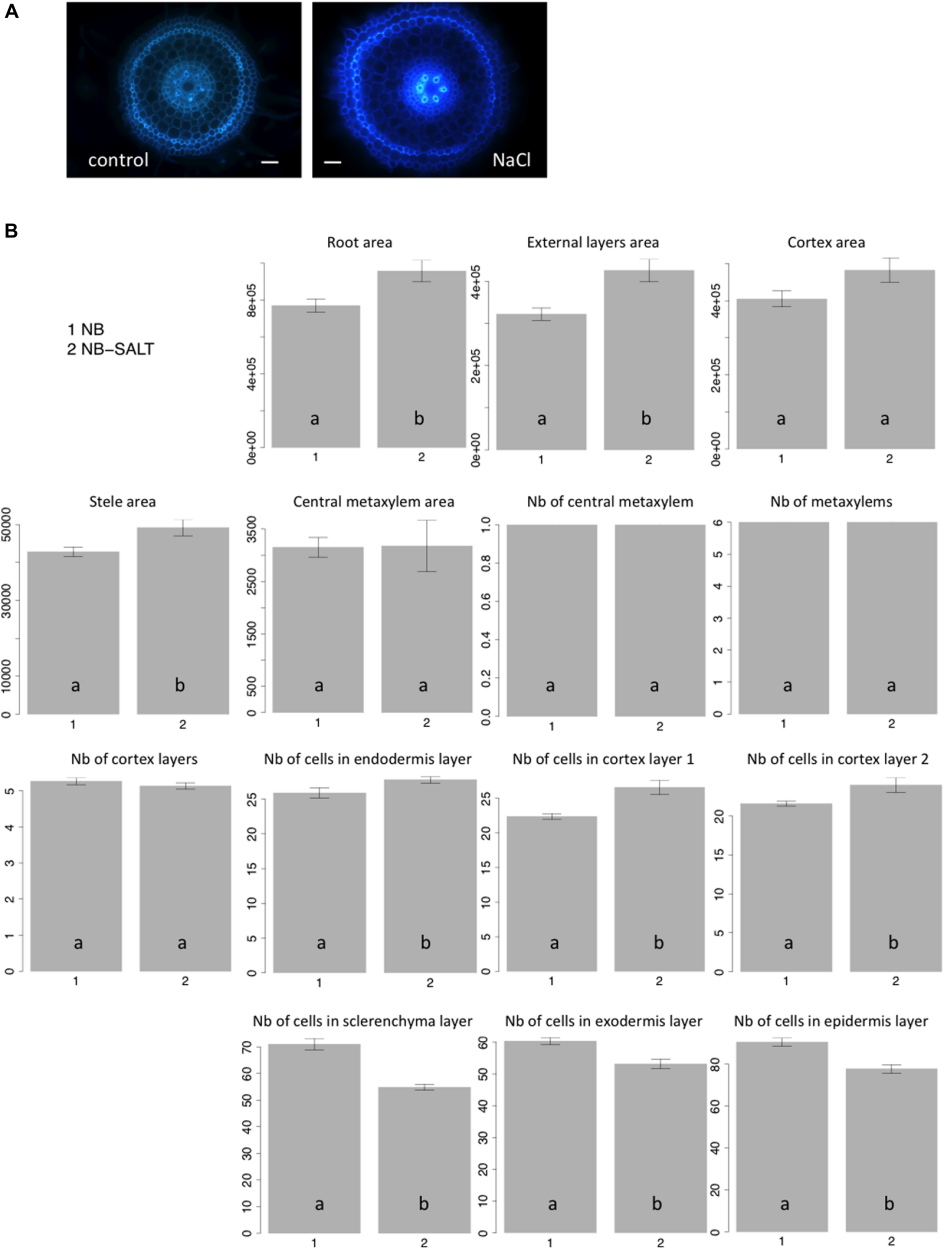
**Nipponbare root anatomical parameters affected by salt stress. (A)** Transverse root sections of Nipponbare seedlings grown on MS/2 medium (Control) and MS/2 medium supplemented with 120 mM NaCl (NaCl); **(B)** Mean values of 10 pictures of Nipponbare roots grown on control medium (NB: 1) and Nipponbare roots grown on NaCl medium (NB-SALT: 2) for each of the parameters measured (root, external layers, cortex, stele, and central metaxylem areas (in pixels); number of central metaxylem, metaxylems, and cortex layers; number of cells in the endodermis, cortex layers 1 and 2, sclerenchyma, exodermis, and epidermis layers). For statistical significance of the data, a Newman–Keuls test has been performed (letters a and b on the histograms).

Salt stress severely affects plant growth and development (Munns and Tester, 2008). Plantlets exposed to salt display adaptive root architectural and anatomical changes (Dinneny, 2010; Galvan-Ampudia and Testerink, 2011). In *Arabidopsis*, salt stress results in radial swelling of the root cortex cells (Burssens et al., 2000; Dinneny et al., 2008). In maize, swelling of the stele tissue has also been described (Li et al., 2014). Ten seedlings of the Nipponbare variety were grown in control versus salt-stress conditions (120 mM), and transverse sections of radicles were photographed after 6 days of growth (**Figure 4A**). Consistent with previous observations of other plants, when grown on NaCl medium, rice radicles show swelling of the roots. Our results showed that the external layers, the cortex and stele areas increased under salt stress (**Figure 4B**). Interestingly, we observed that the cell number per cell file was higher in all internal tissues, including endodermis and cortex, under salt stress. By contrast, the number of cells in external cell files (sclerenchyma, exodermis, and epidermis) was reduced. Our results show that the swelling of the external root tissues is due to an increase of cell area while cell division is inhibited. Interestingly, salt stress induces an increase of cell division in internal cell layers.

## CONCLUSION

The PHIV-RootCell toolset is a simple and robust tool well-adapted to quantifying root anatomical trait variation. We have demonstrated that this software is compatible with the study of genetic diversity and the influence of environmental constraints on internal structures of rice roots. This tool will be utilized in the future for QTL and GWAS analyses and to evaluate physiological responses of rice roots to various conditions.

## AUTHOR CONTRIBUTIONS

Marc Lartaud, Christophe Perin, Emilie Thomas, Anne Dievart: conception and design of the PHIV-RootCell toolset; Emilie Thomas, Sophia Henry, Mathilde Bettembourg, Fanchon Divol, Nadege Lanau, Florence Artus, Charlotte Bureau, Anne Dievart: acquisition and analysis of data; Christophe Perin, Brigitte Courtois, Anne Dievart: interpretation of data; Gautier Sarah, Anne Dievart: conception and design of the PHIV-RootCell R script; Christophe Perin, Brigitte Courtois, Jean-Luc Verdeil, Emmanuel Guiderdoni, Anne Dievart: drafting of the manuscript.

## SUPPLEMENTARY MATERIAL

The Supplementary Material for this article can be found online at: http://www.frontiersin.org/journal/10.3389/fpls.2014.00790/abstract

## Conflict of Interest Statement

The authors declare that the research was conducted in the absence of any commercial or financial relationships that could be construed as a potential conflict of interest.

## Footnotes

1

2

3
