# Supplementary figures and images for: PHIV-RootCell: a supervised image analysis tool for rice root anatomical parameter quantification
Source: Front Plant Sci. 2015 Jan 19;5:790. doi: 10.3389/fpls.2014.00790 (PMC4298167; doi:10.3389/fpls.2014.00790)

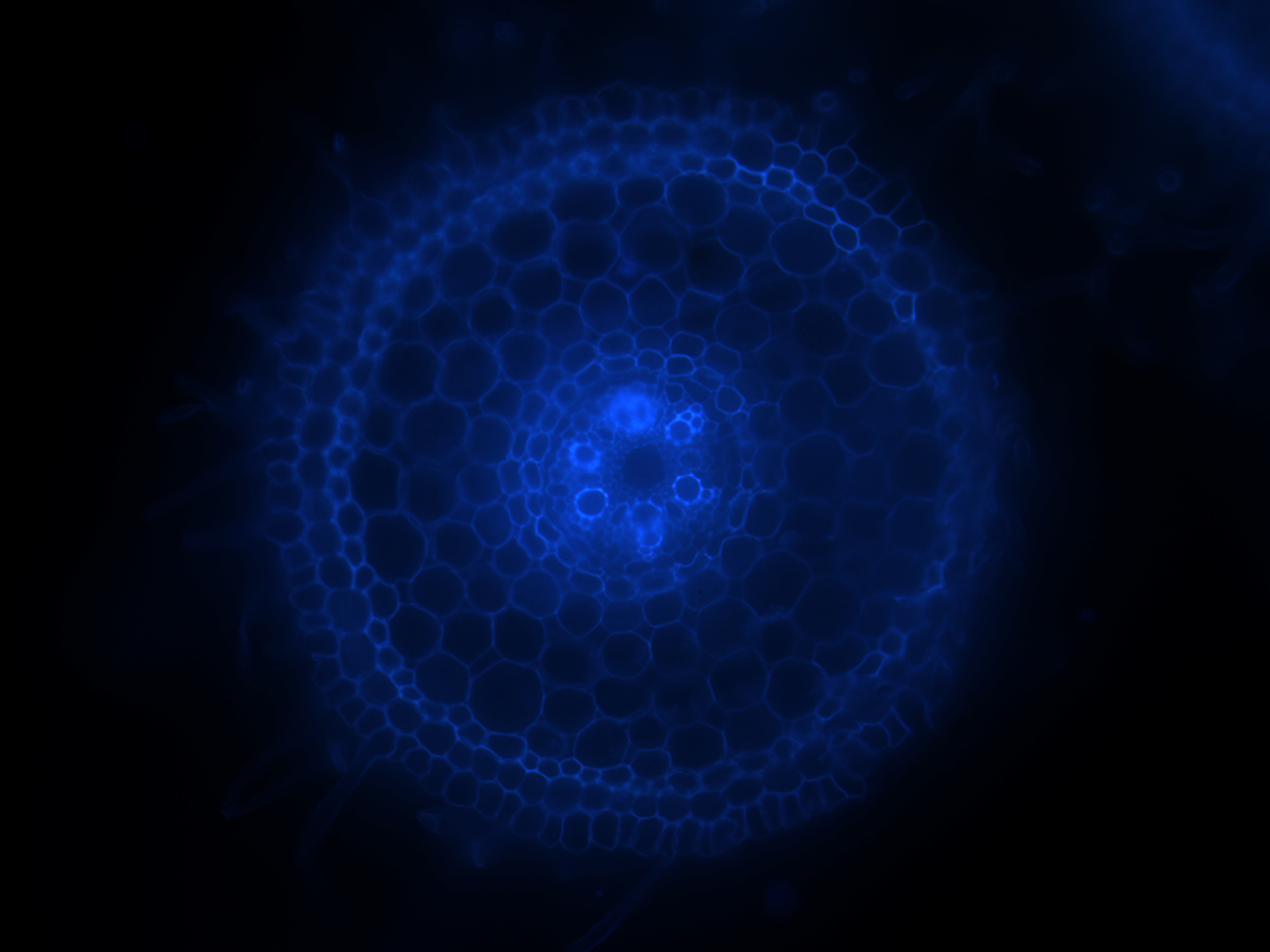

Supplement: Supplementary file 1 [file Images.ZIP › IMAGES/NB1.tif]

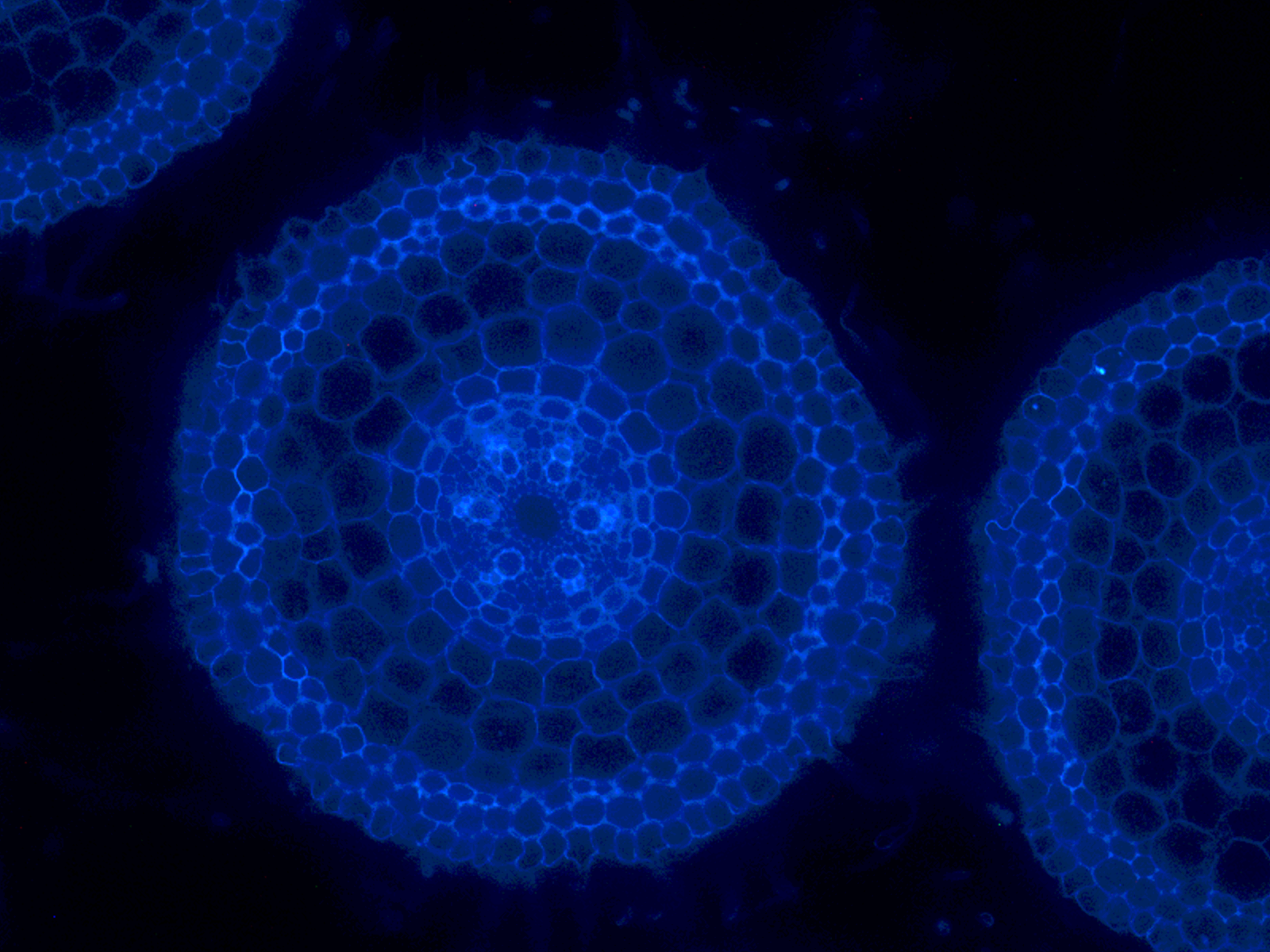

Supplement: Supplementary file 1 [file Images.ZIP › IMAGES/NB10.tif]

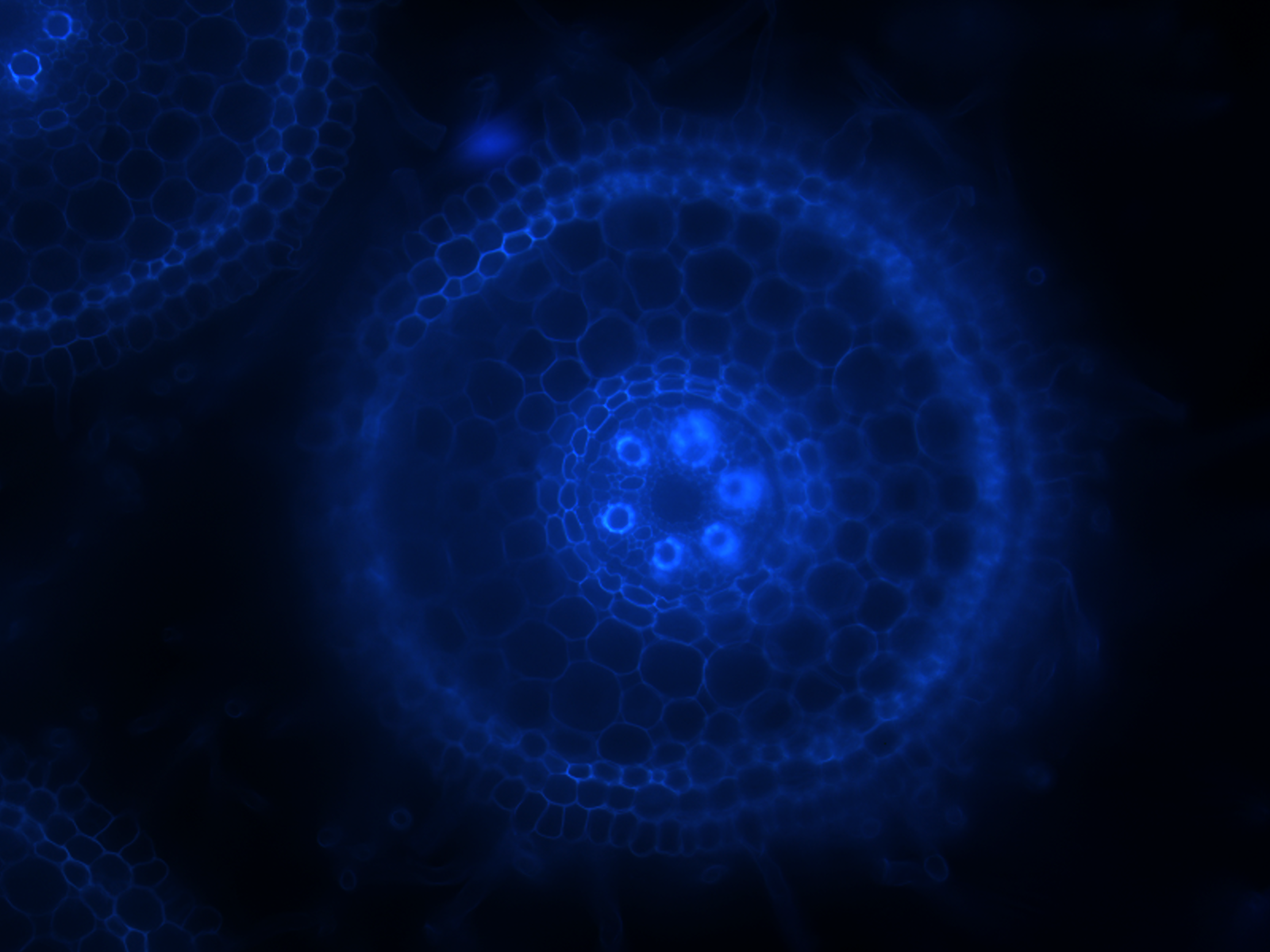

Supplement: Supplementary file 1 [file Images.ZIP › IMAGES/NB2.tif]

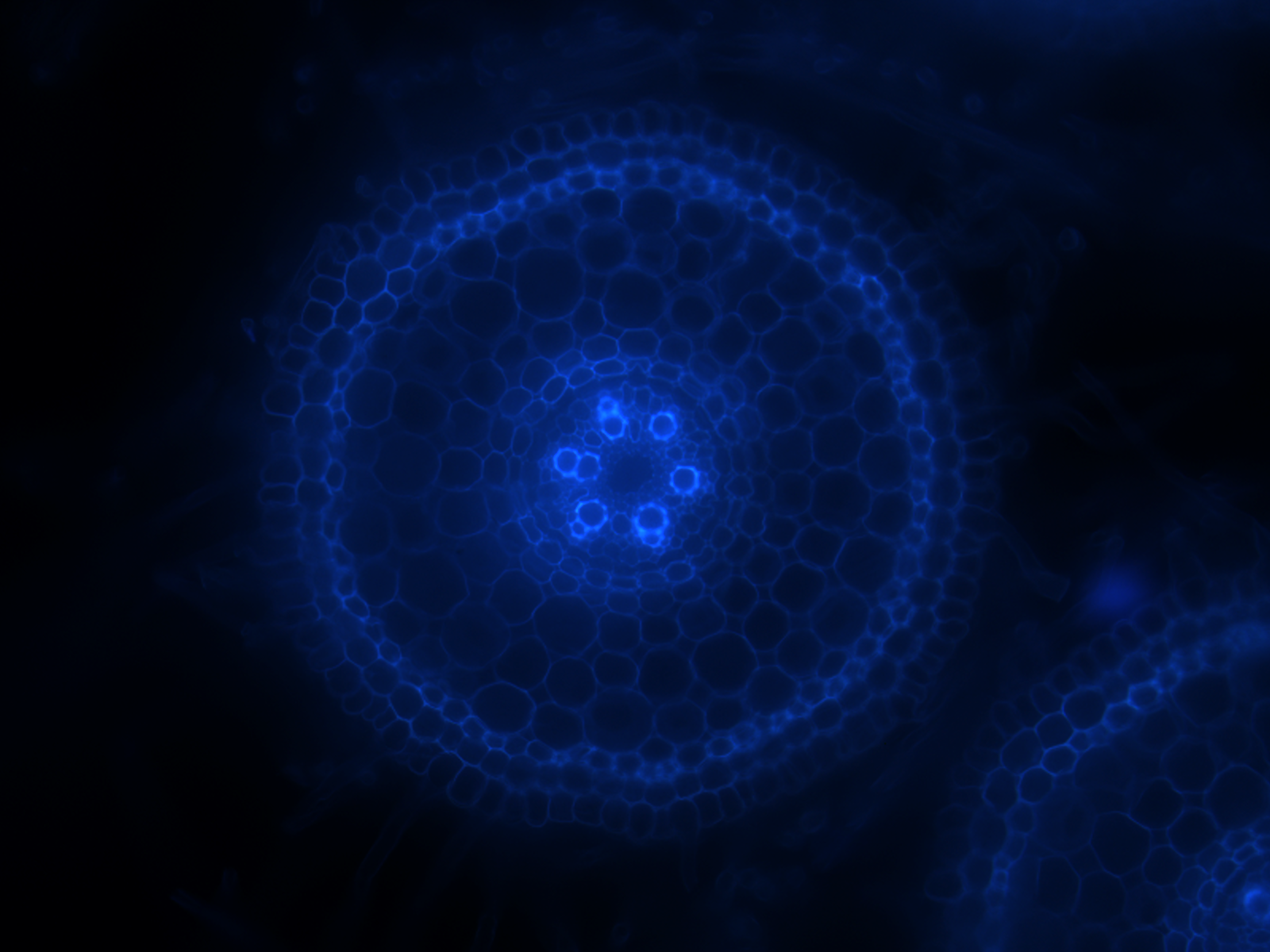

Supplement: Supplementary file 1 [file Images.ZIP › IMAGES/NB3.tif]

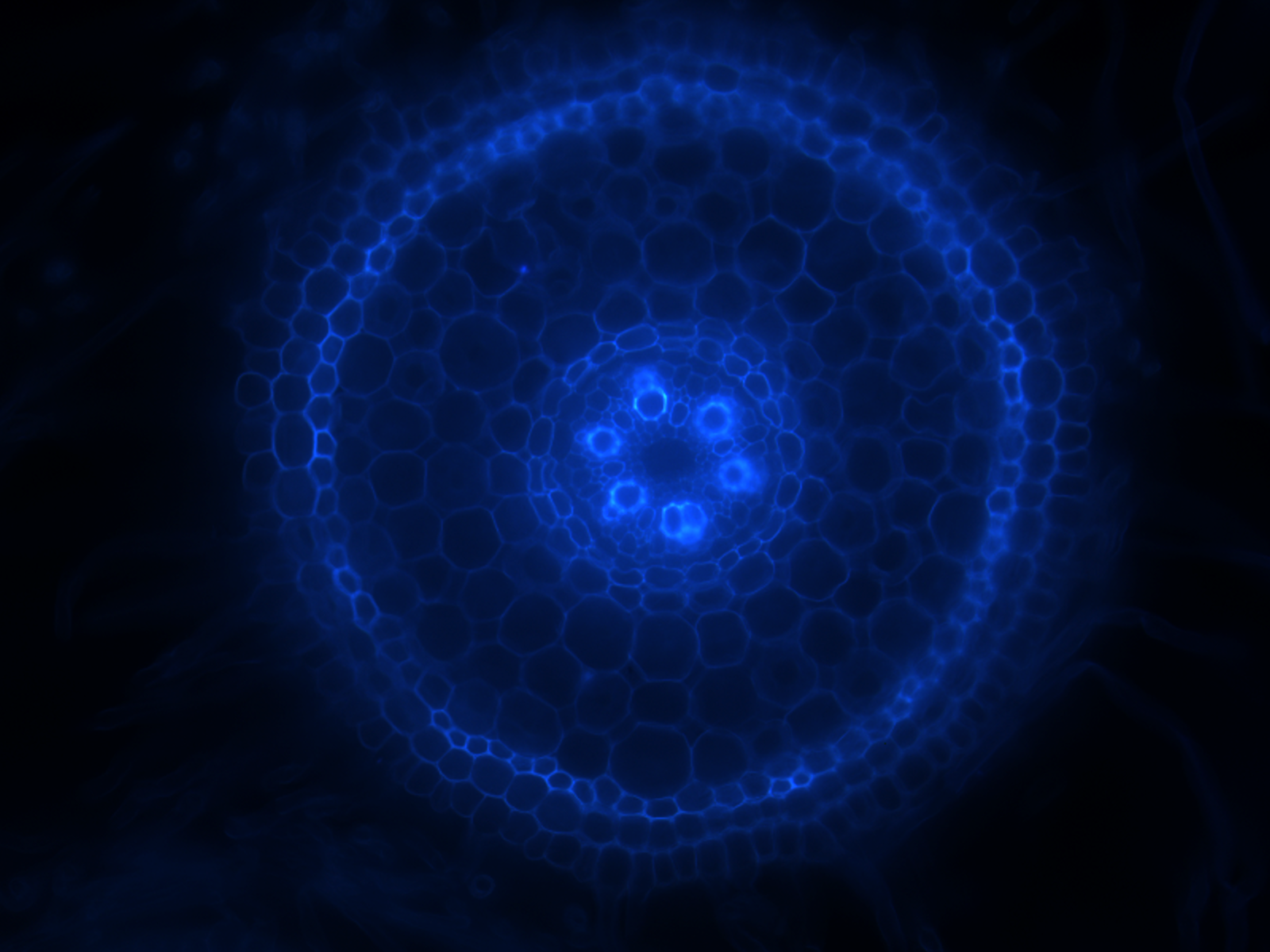

Supplement: Supplementary file 1 [file Images.ZIP › IMAGES/NB4.tif]

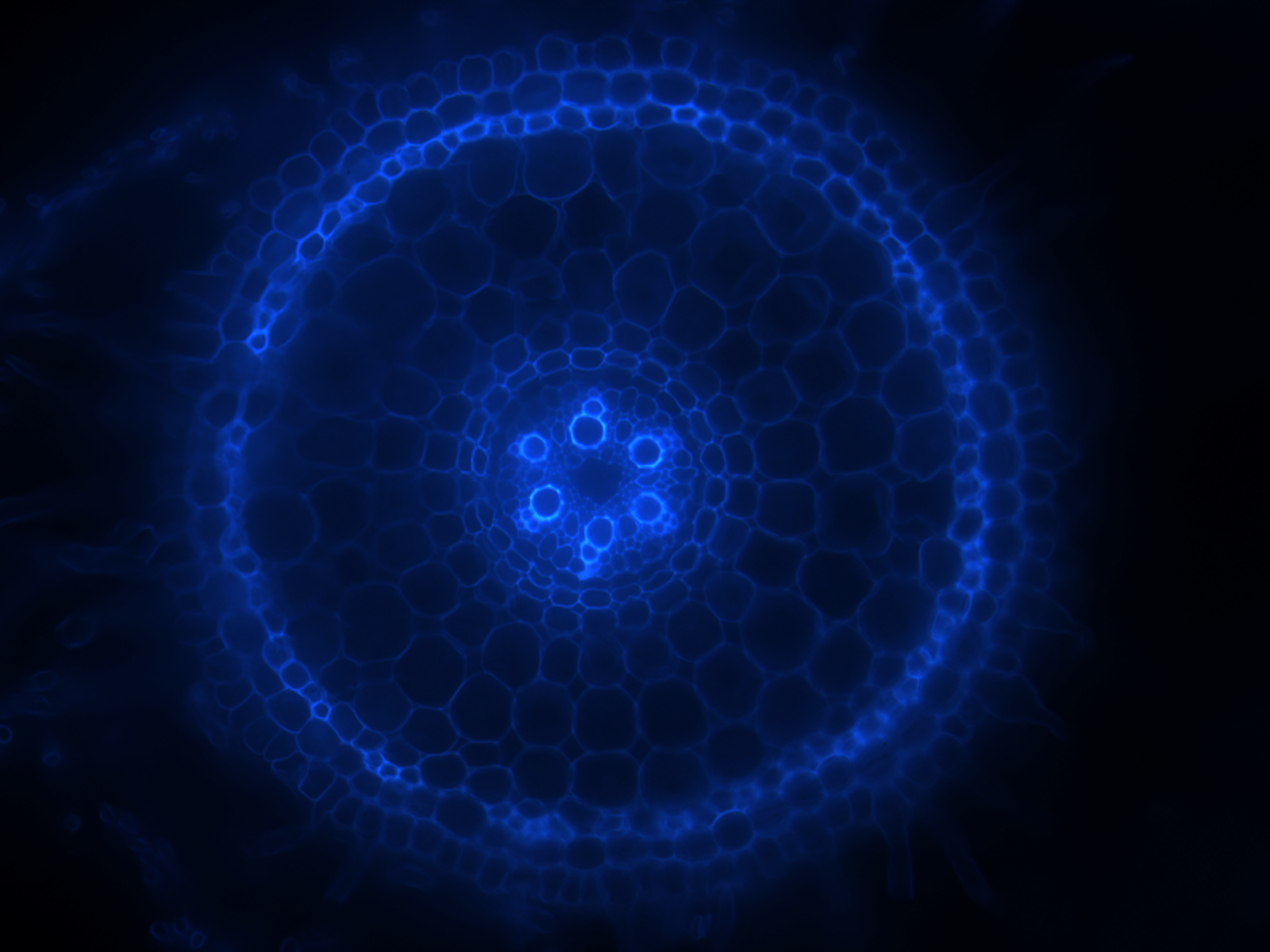

Supplement: Supplementary file 1 [file Images.ZIP › IMAGES/NB5.tif]

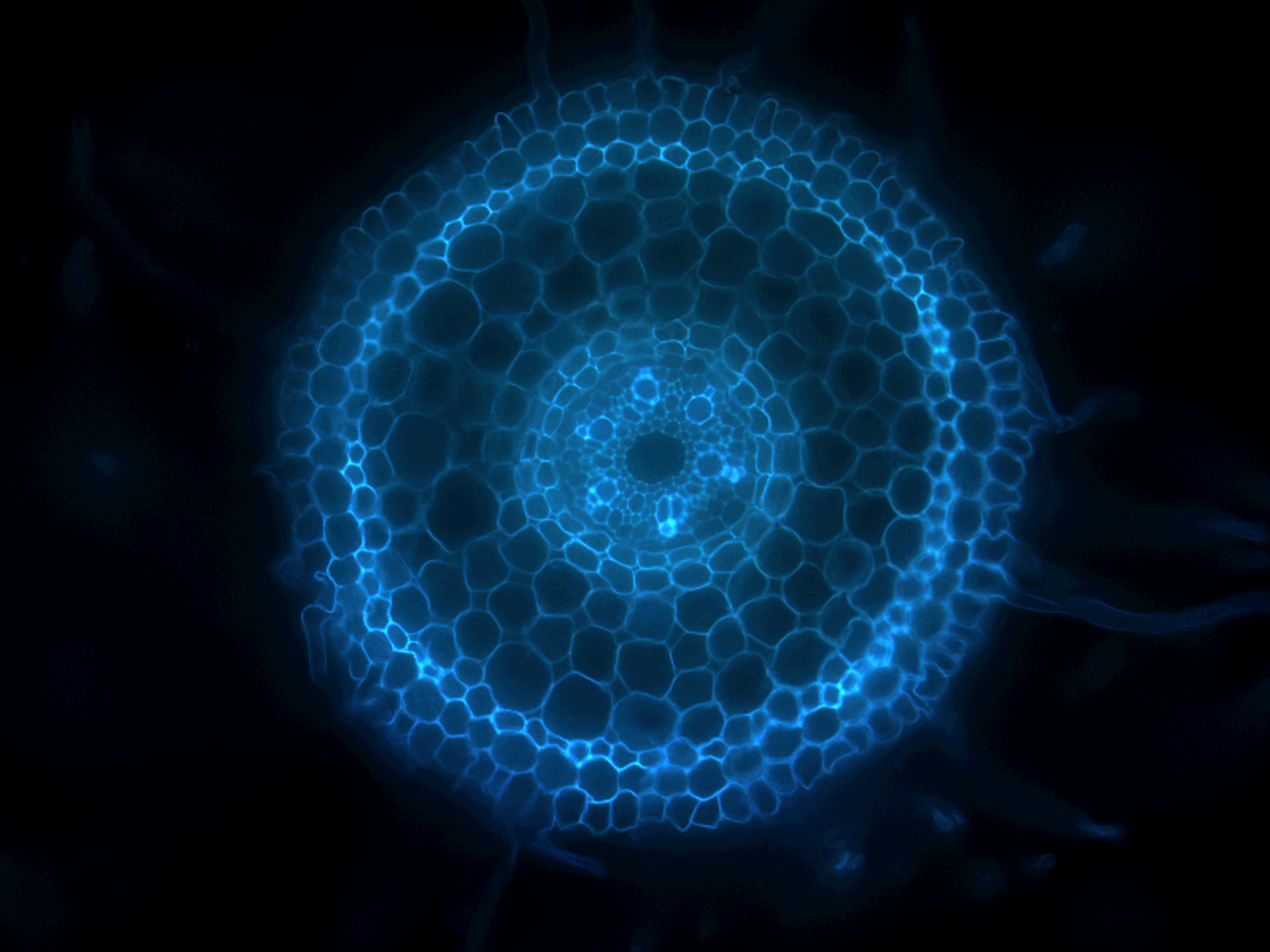

Supplement: Supplementary file 1 [file Images.ZIP › IMAGES/NB6.tif]

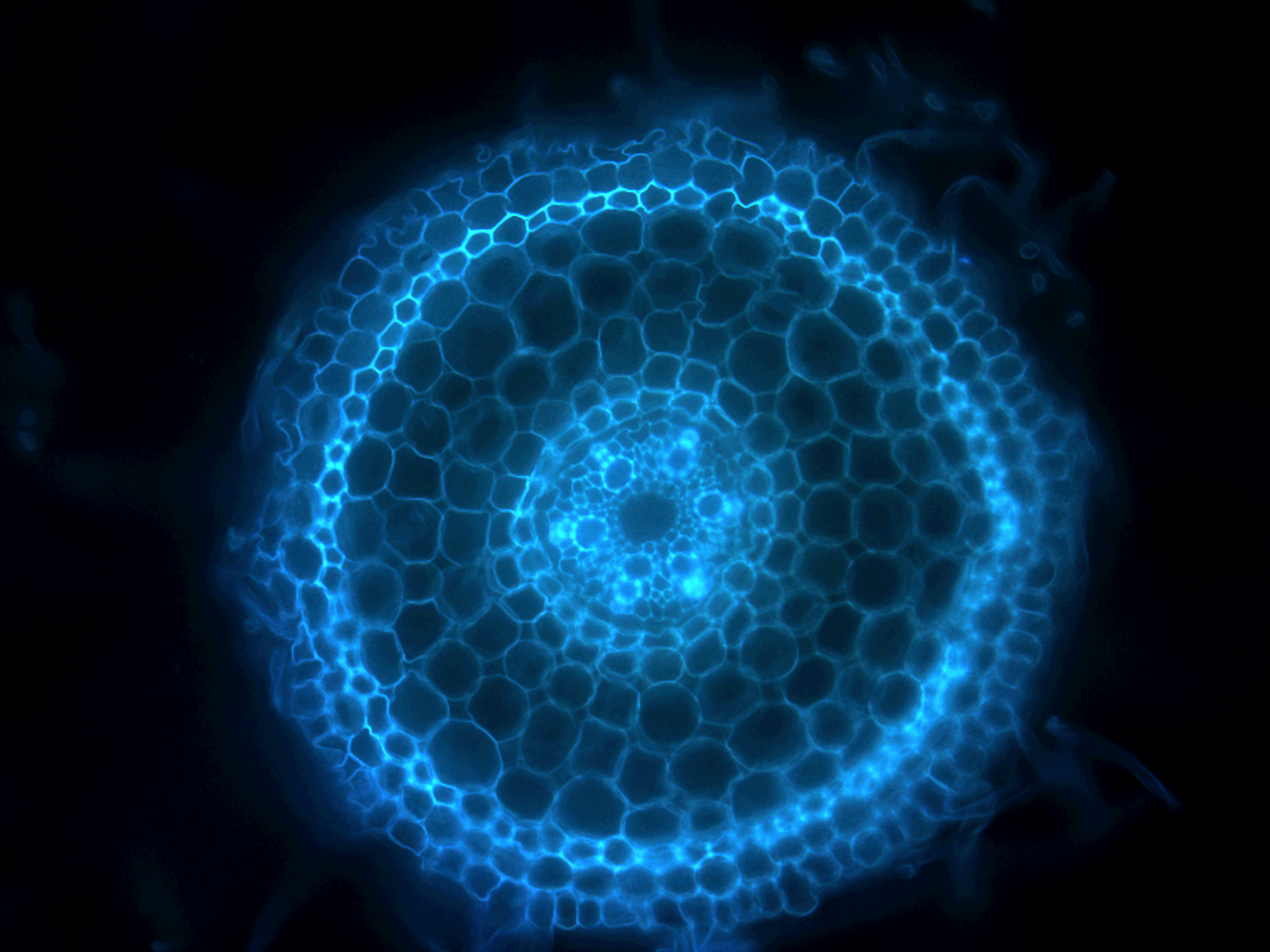

Supplement: Supplementary file 1 [file Images.ZIP › IMAGES/NB7.tif]

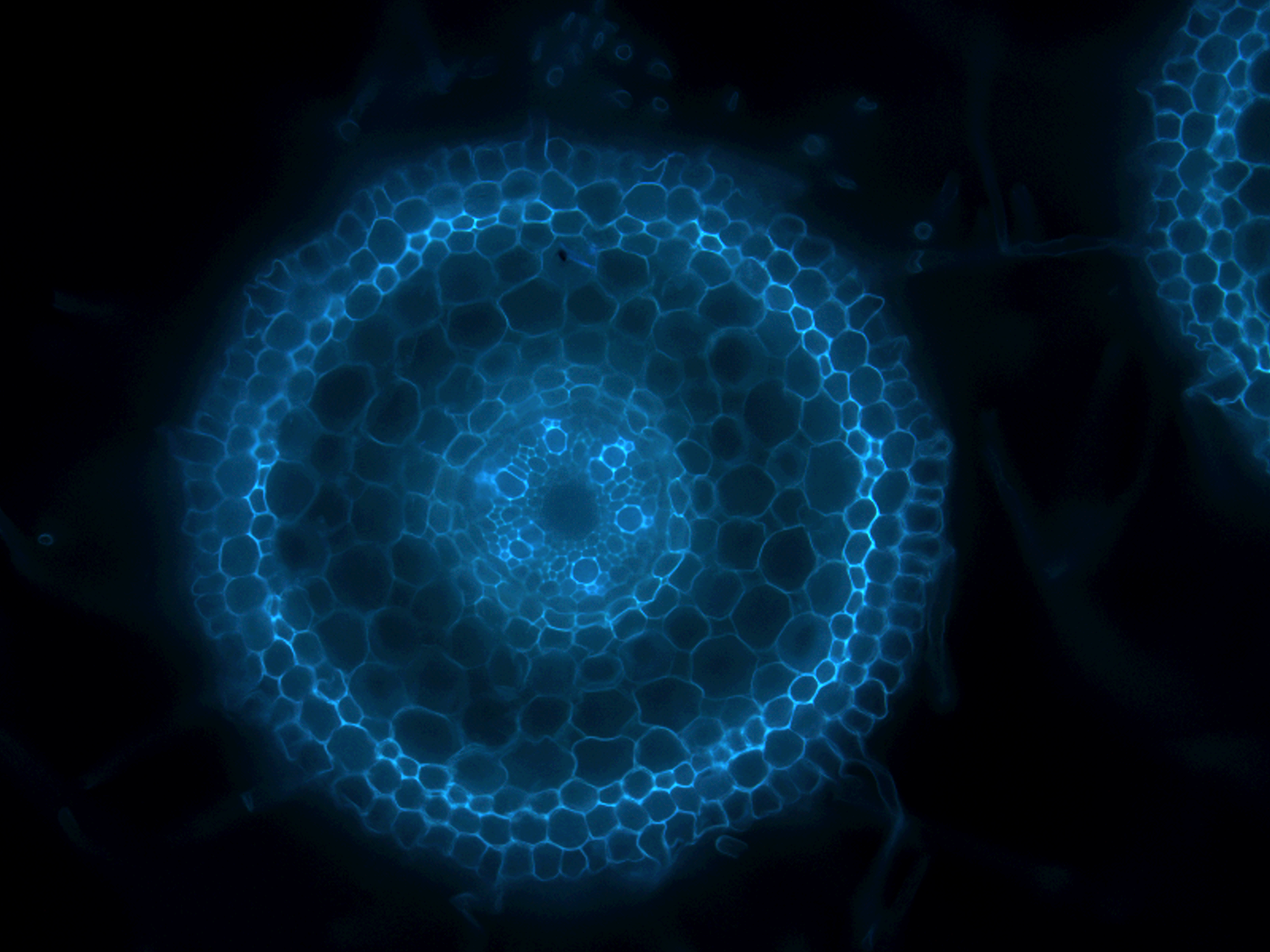

Supplement: Supplementary file 1 [file Images.ZIP › IMAGES/NB8.tif]

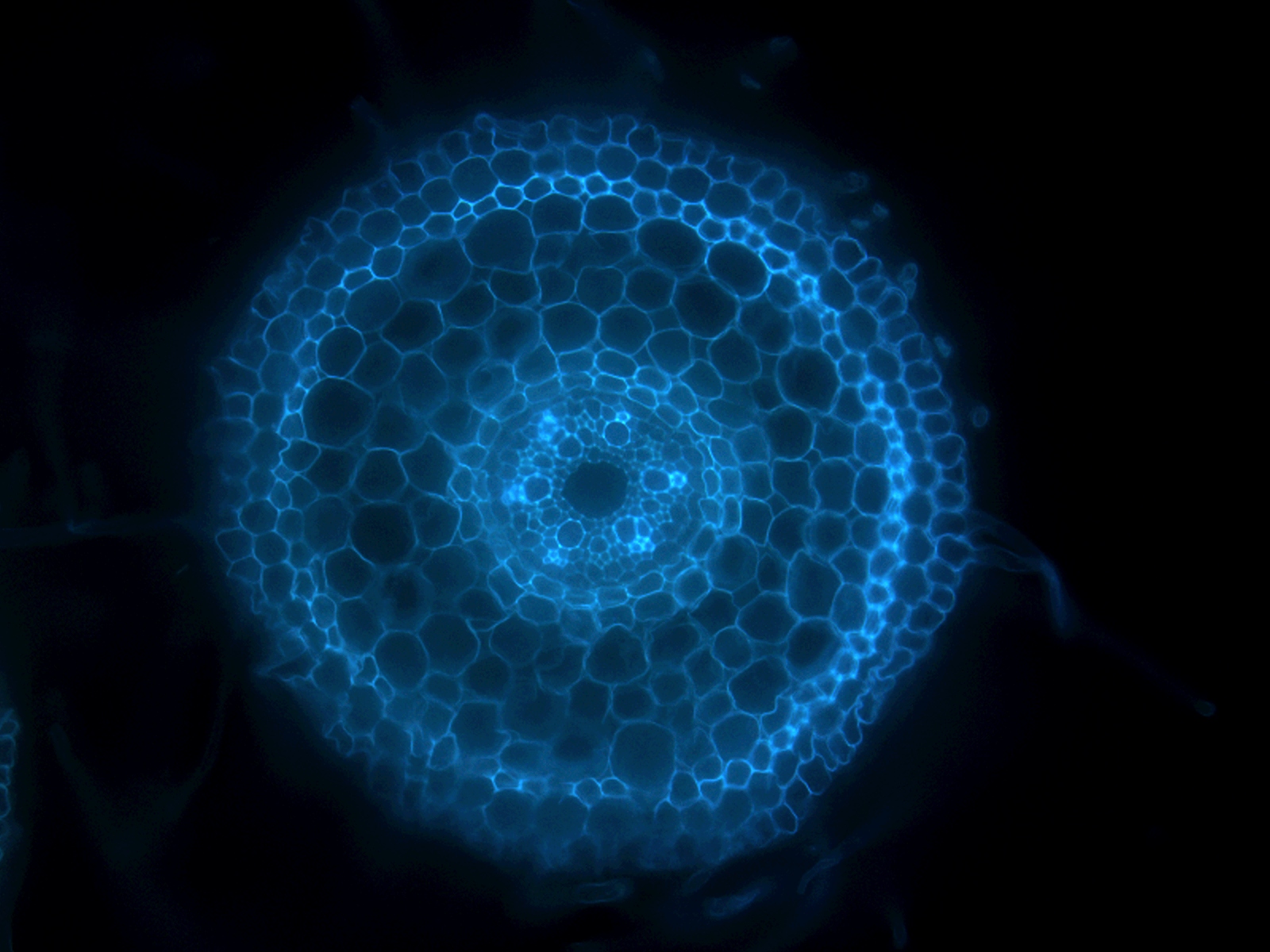

Supplement: Supplementary file 1 [file Images.ZIP › IMAGES/NB9.tif]
