# Supplementary material for: PHIV-RootCell: a supervised image analysis tool for rice root anatomical parameter quantification
Source: Front Plant Sci. 2015 Jan 19;5:790. doi: 10.3389/fpls.2014.00790 (PMC4298167; doi:10.3389/fpls.2014.00790)
Supplement: Supplementary file 3 [file R-files.ZIP › R-files/Analyses.pdf]

Analyses.pdf

1 1_ASD 1
2 1_GAMBIAKA
3 1_KHAO DAWK MALI 105
4 1_TEQUING
5 2_FR13A
6 2_N 22
7 3_BAMOIA 341
8 4_RAYADA
9 5_KAUKKYI ANI
10 6−temp_GIZA 171
11 6−temp_M 202
12 6−temp_NIPPONBARE
13 6−Trop_AZUCENA
14 6−Trop_GOGO LEMPAK
15 6−Trop_IAC 165
16 6−Trop_KARASUKARA SURANKASU

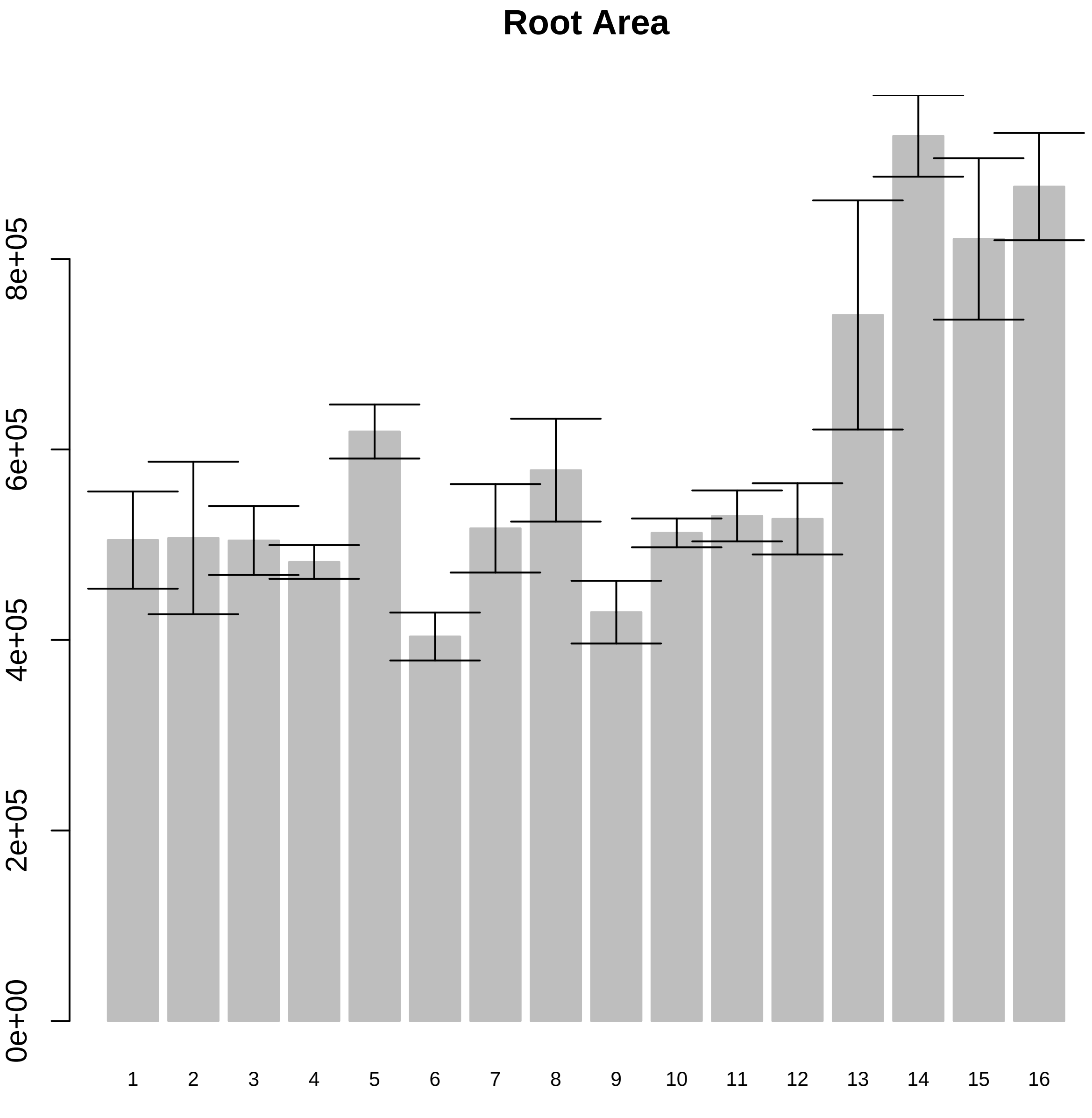

Root Area
0e+00
2e+05
4e+05
6e+05
8e+05
1
2
3
4
5
6
7
8
9
10
11
12
13
14
15
16

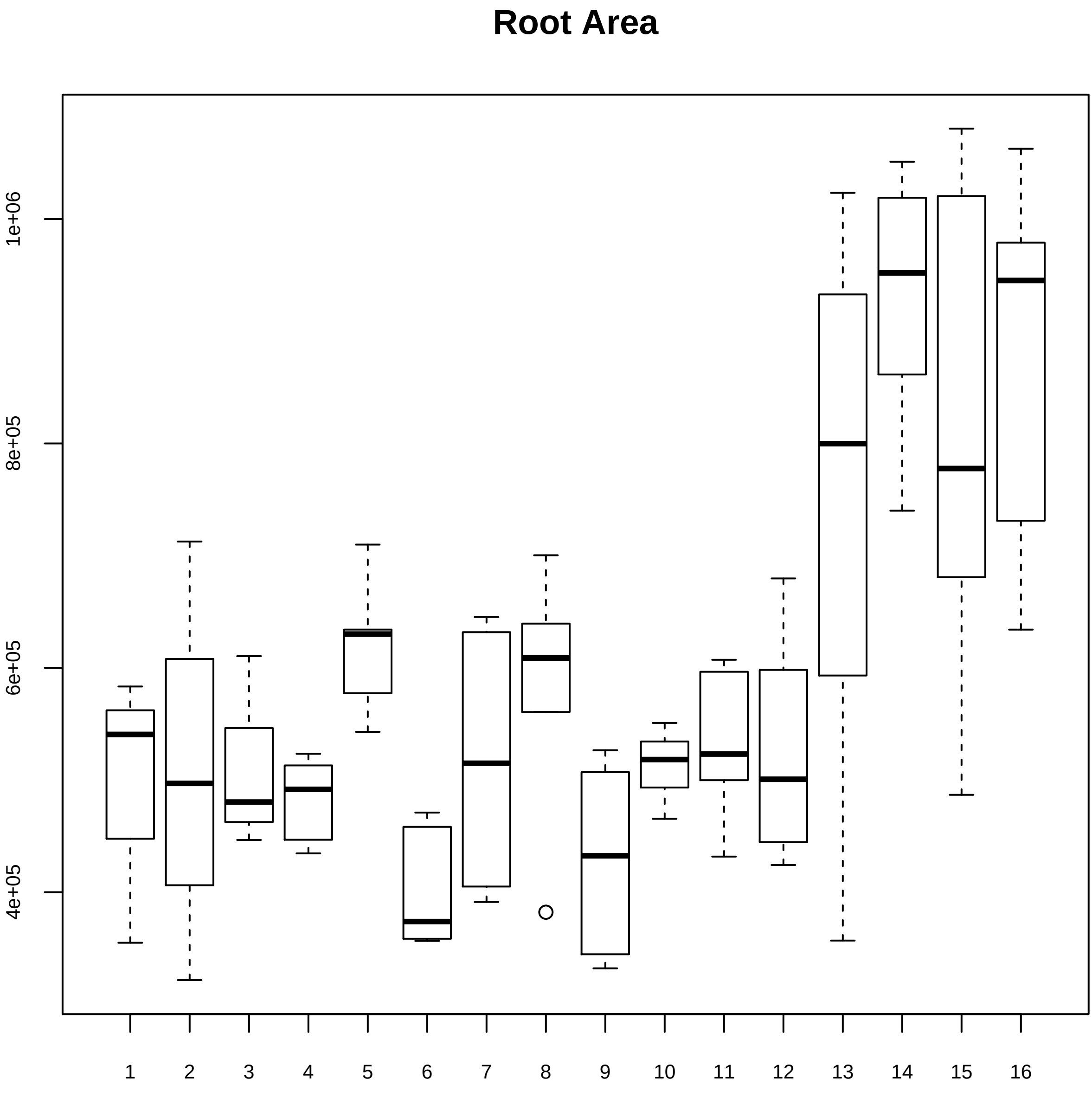

Root Area
1e+06
8e+05
6e+05
4e+05
1
2
3
4
5
6
7
8
9
10
11
12
13
14
15
16

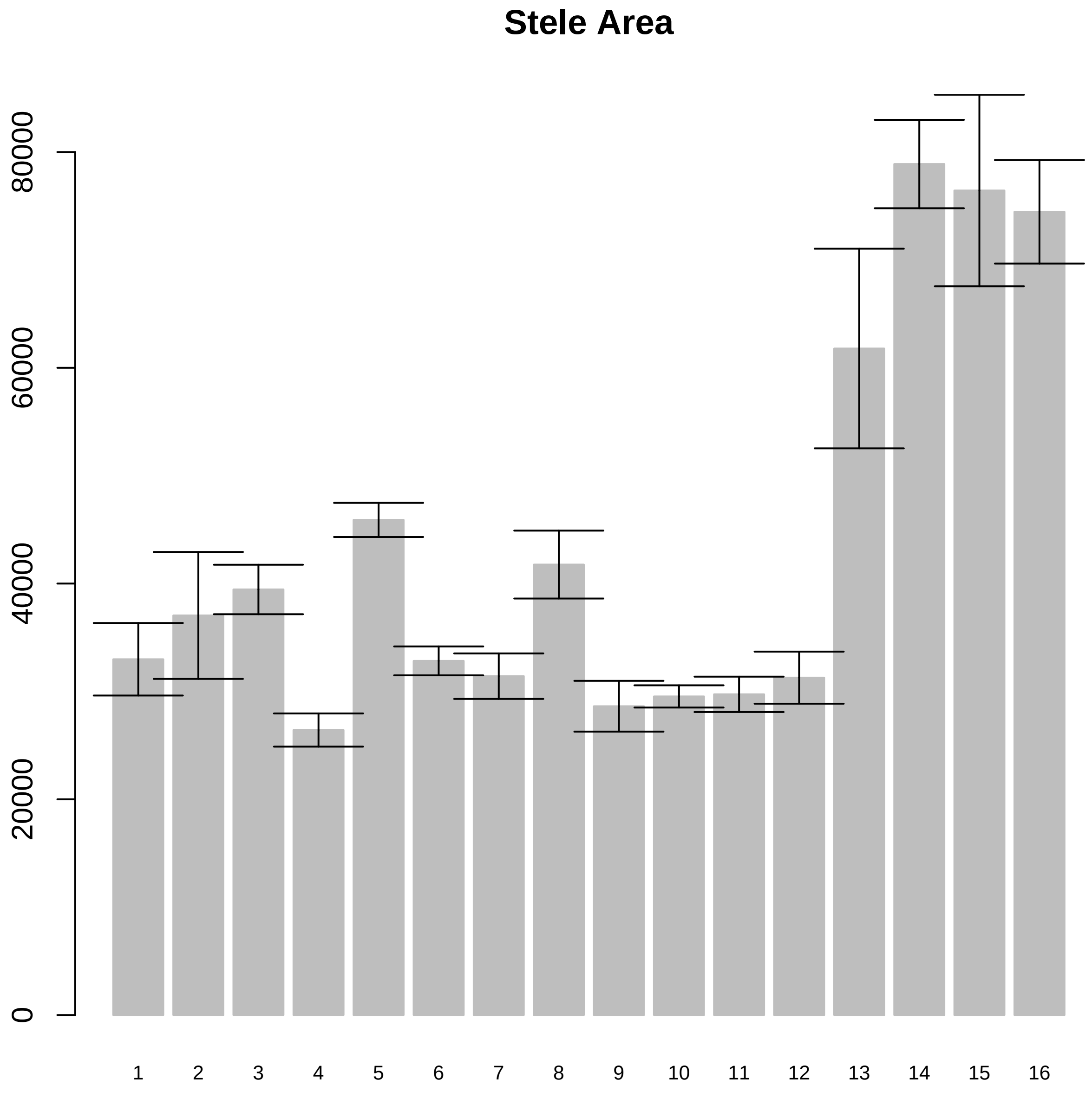

Stele Area
0
20000
40000
60000
80000
1
2
3
4
5
6
7
8
9
10
11
12
13
14
15
16

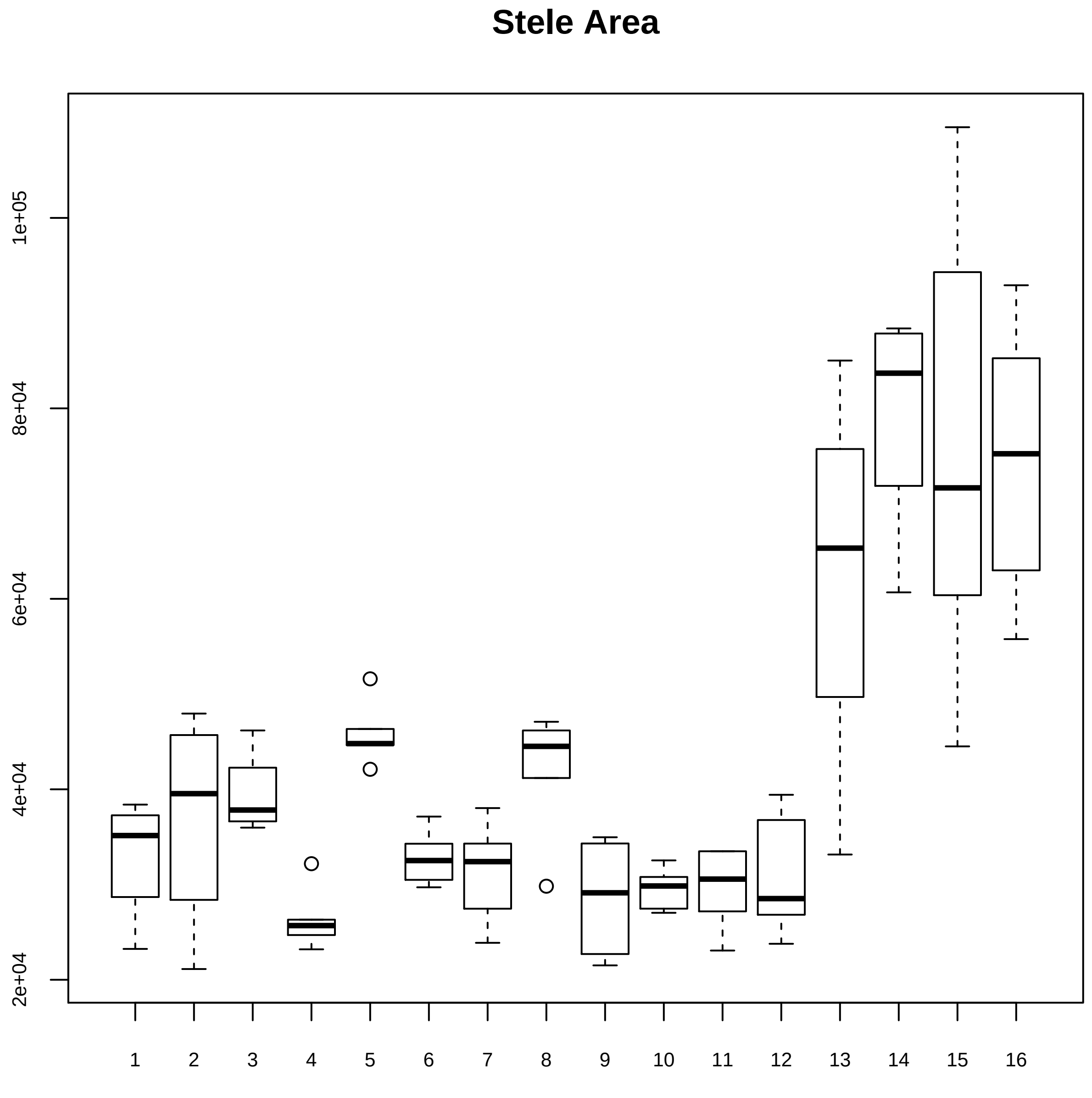
Stele Area
1e+05
8e+04
6e+04
4e+04
2e+04
1
2
3
4
5
6
7
8
9
10
11
12
13
14
15
16

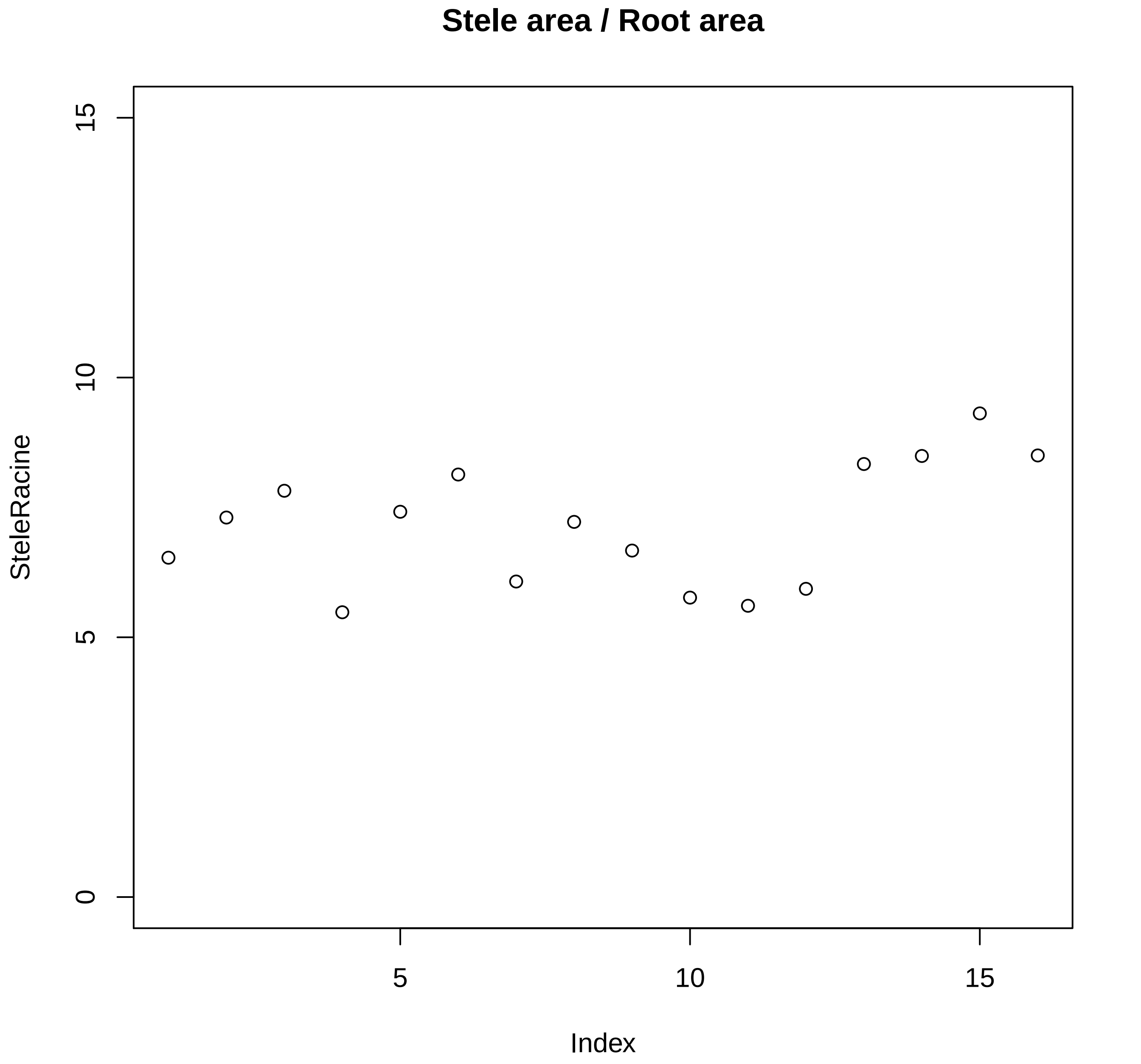
Stele area / Root area
SteleRacine
Index
0
5
10
15
5
10
15

External Layer Area

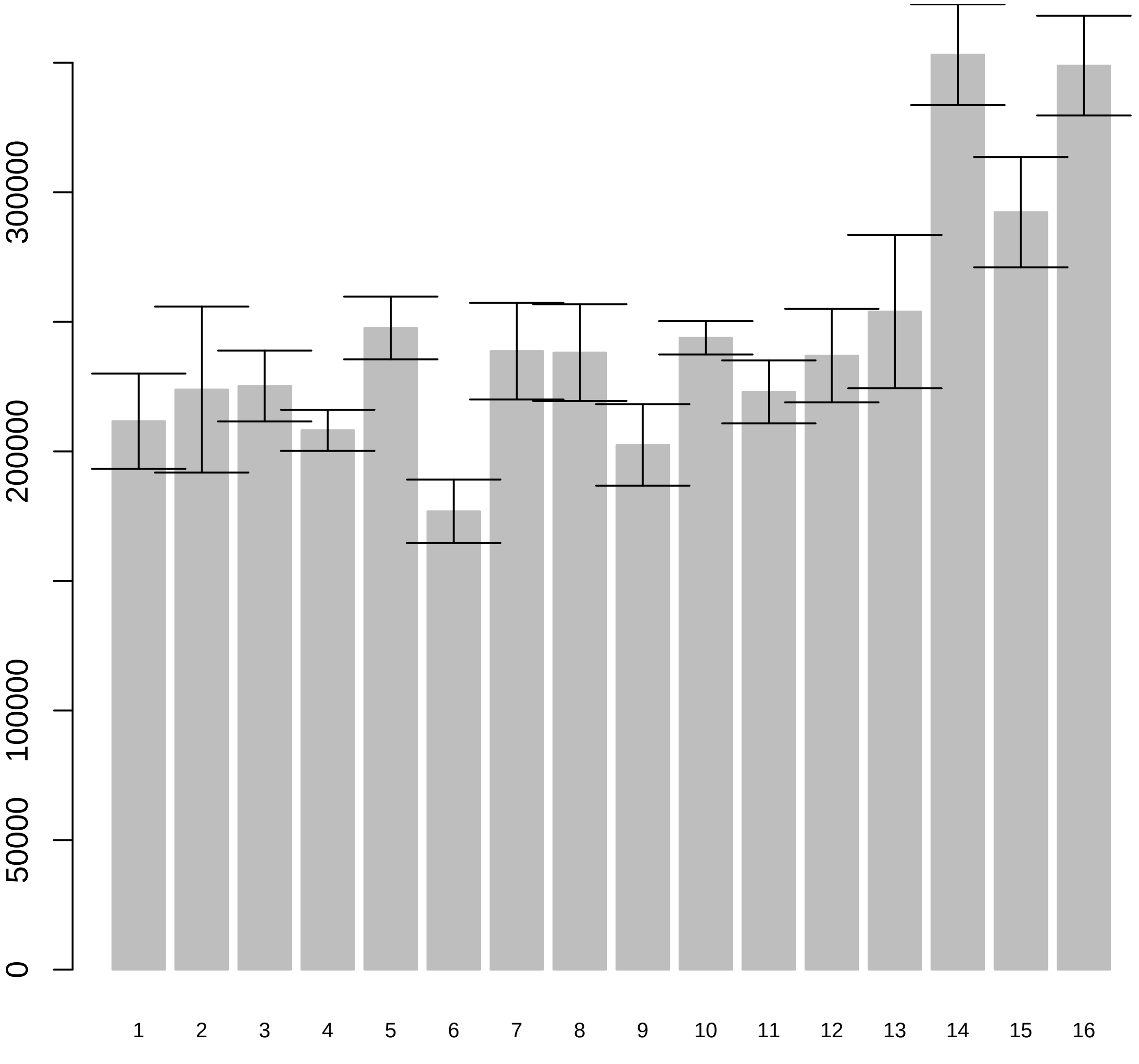

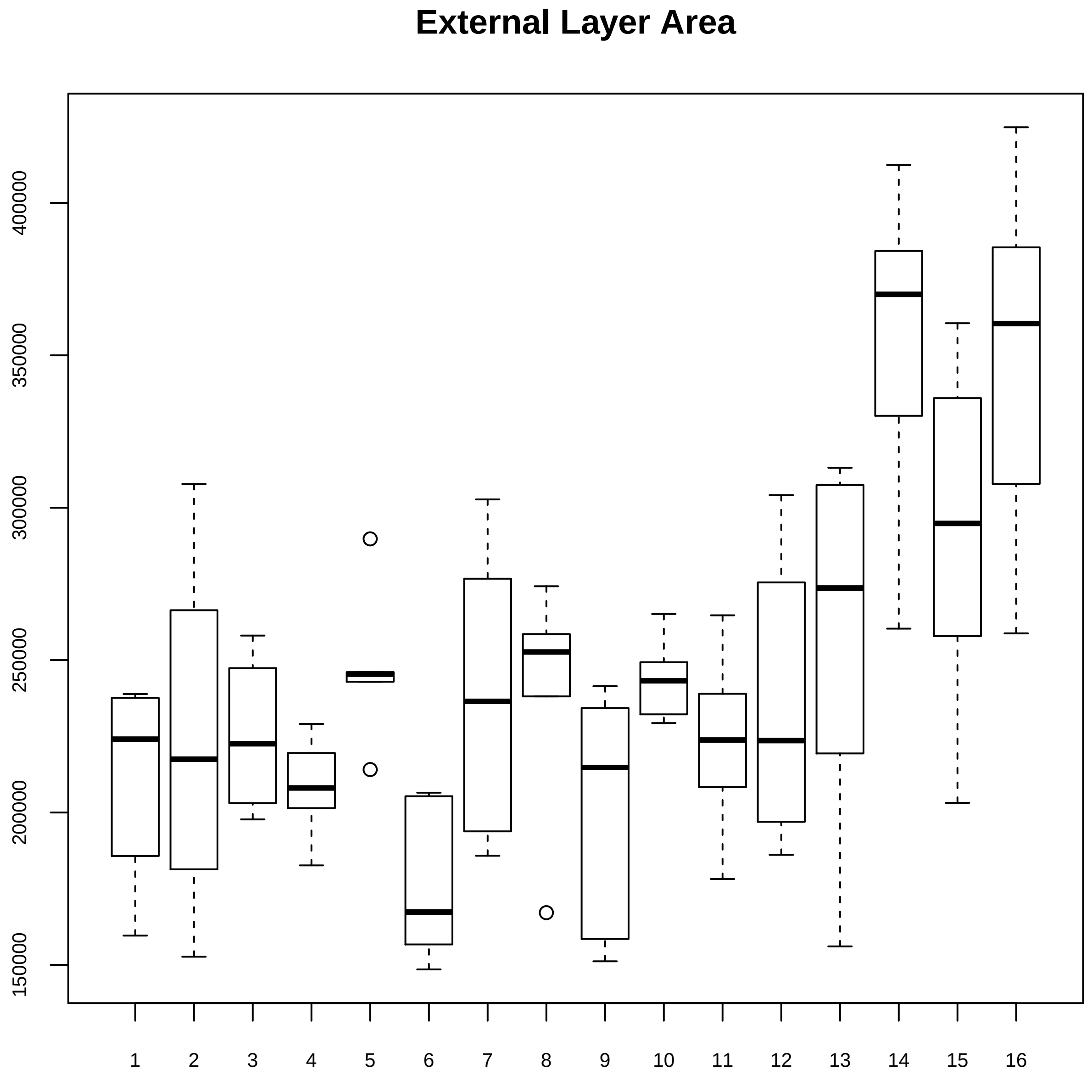
External Layer Area
150000
200000
250000
300000
350000
400000
1
2
3
4
5
6
7
8
9
10
11
12
13
14
15
16

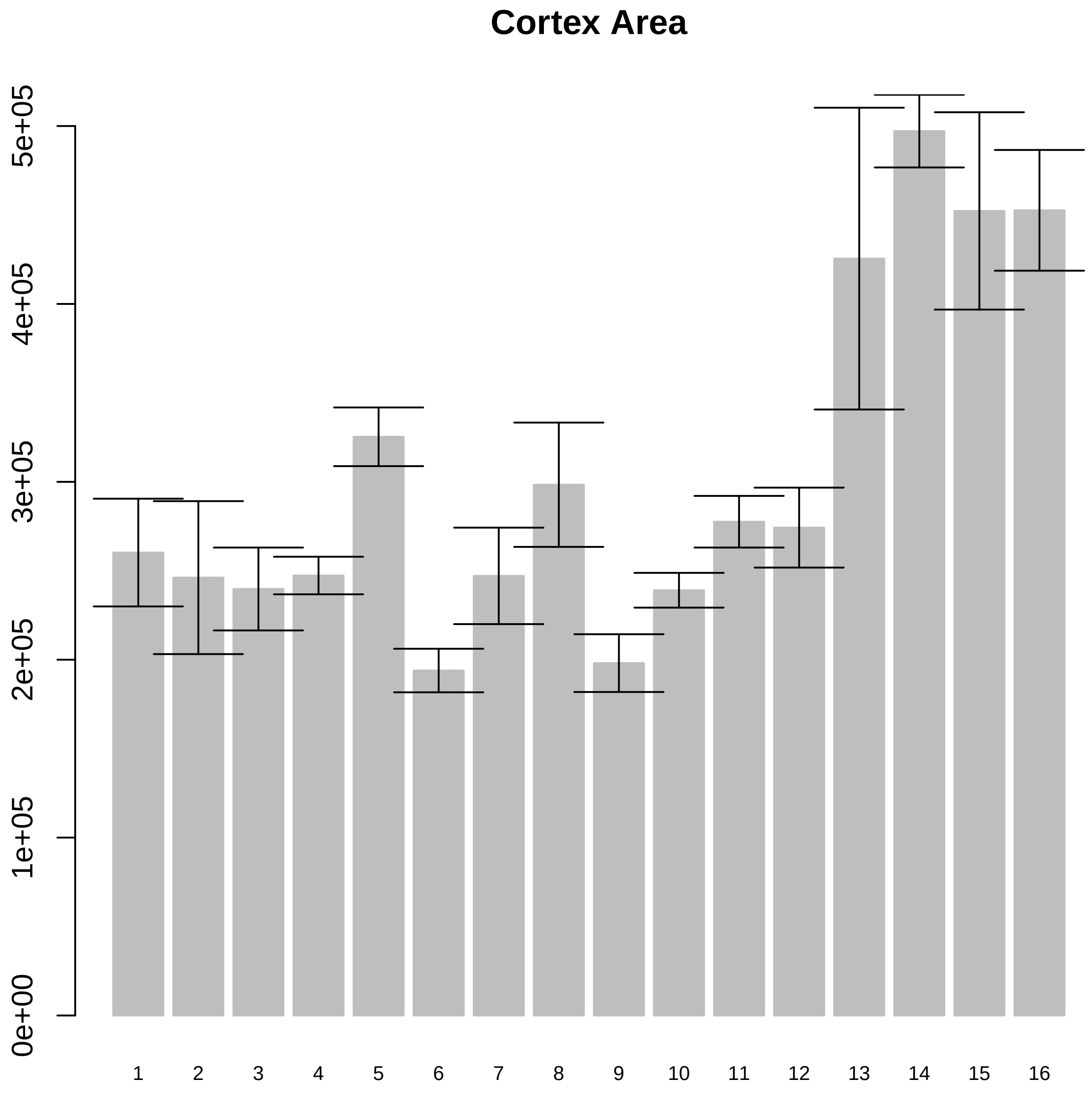

Cortex Area
0e+00
1e+05
2e+05
3e+05
4e+05
5e+05
1
2
3
4
5
6
7
8
9
10
11
12
13
14
15
16

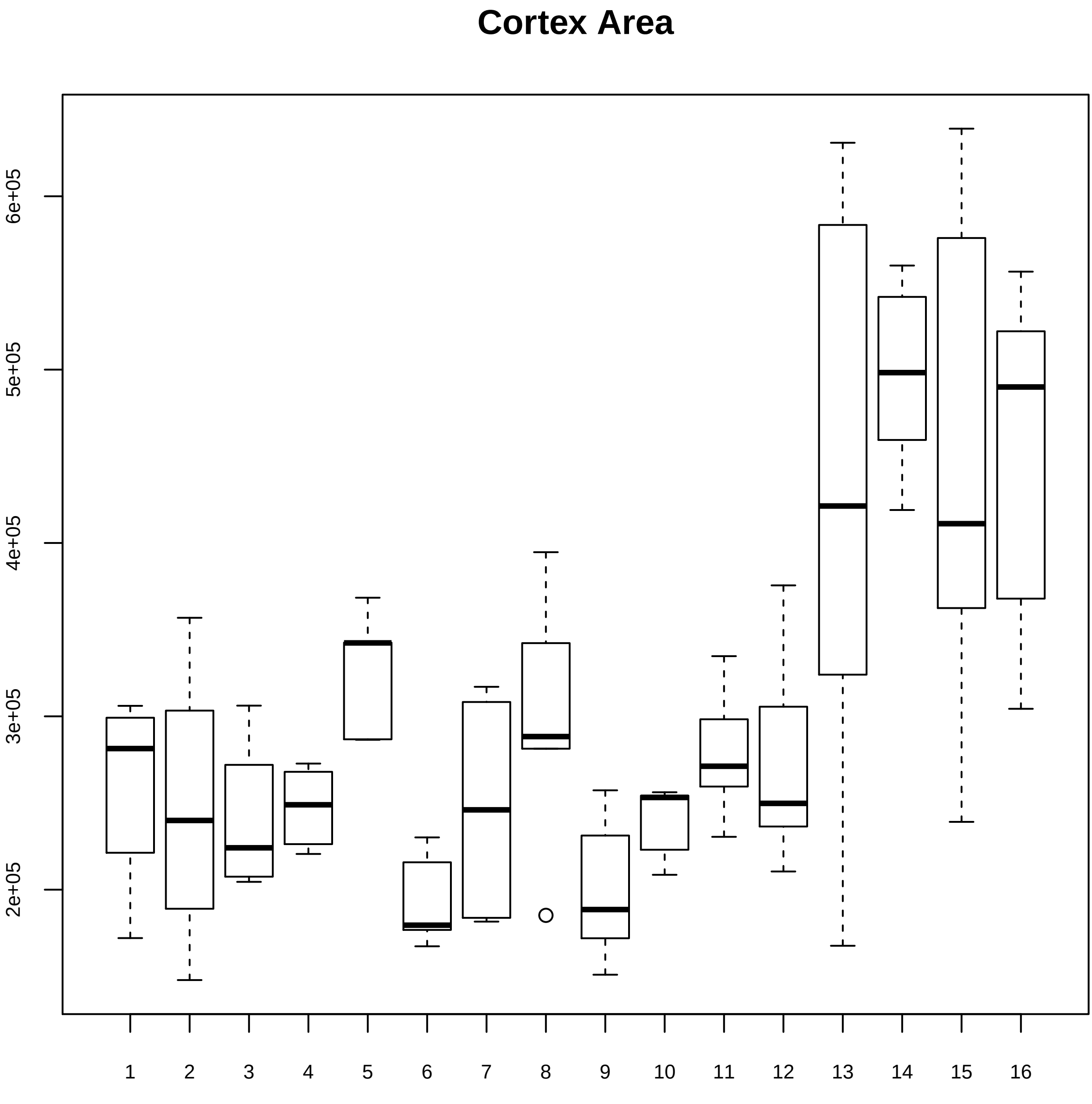

Cortex Area
6e+05
5e+05
4e+05
3e+05
2e+05
1
2
3
4
5
6
7
8
9
10
11
12
13
14
15
16

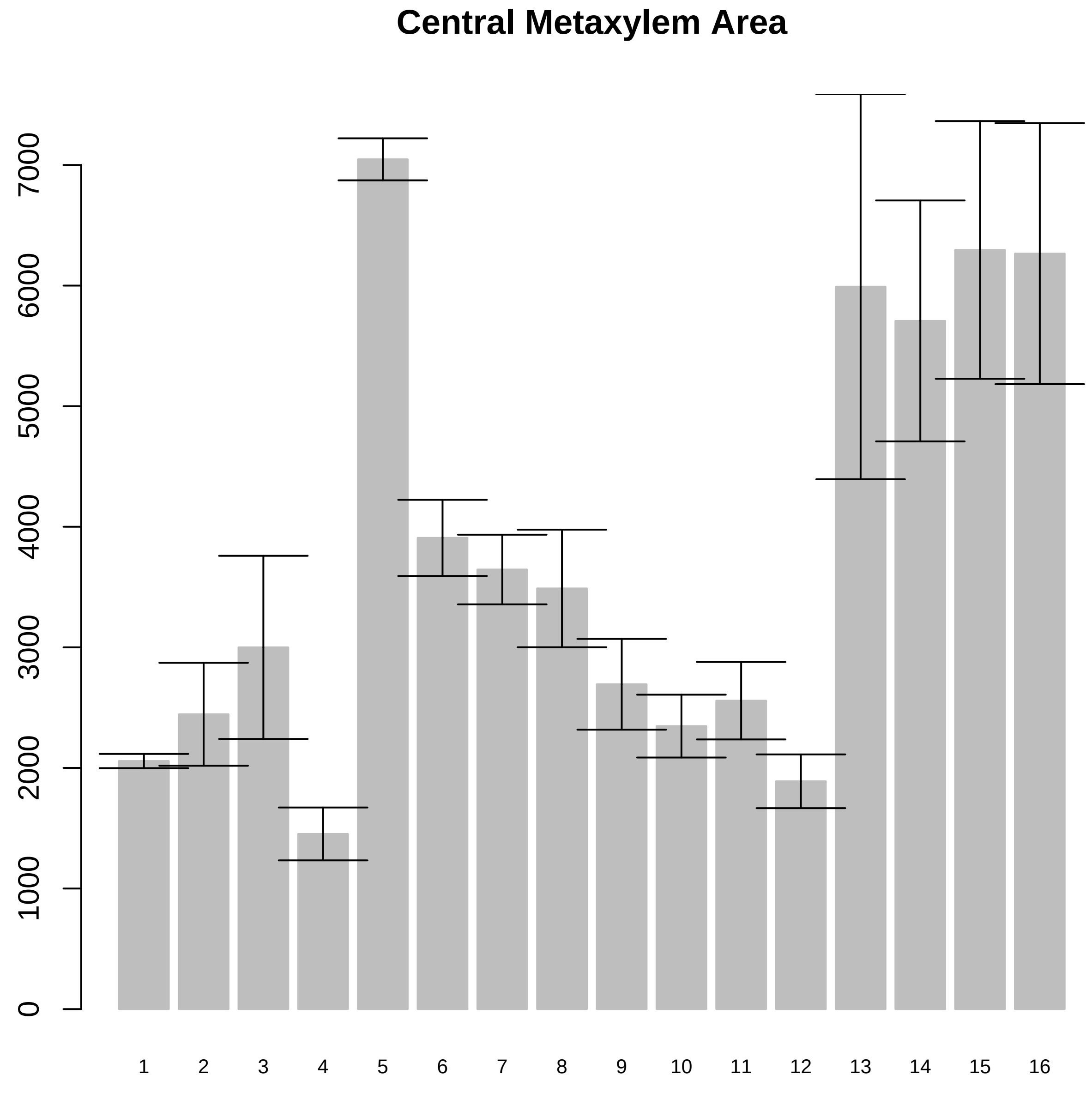

Central Metaxylem Area
0
1000
2000
3000
4000
5000
6000
7000
1
2
3
4
5
6
7
8
9
10
11
12
13
14
15
16

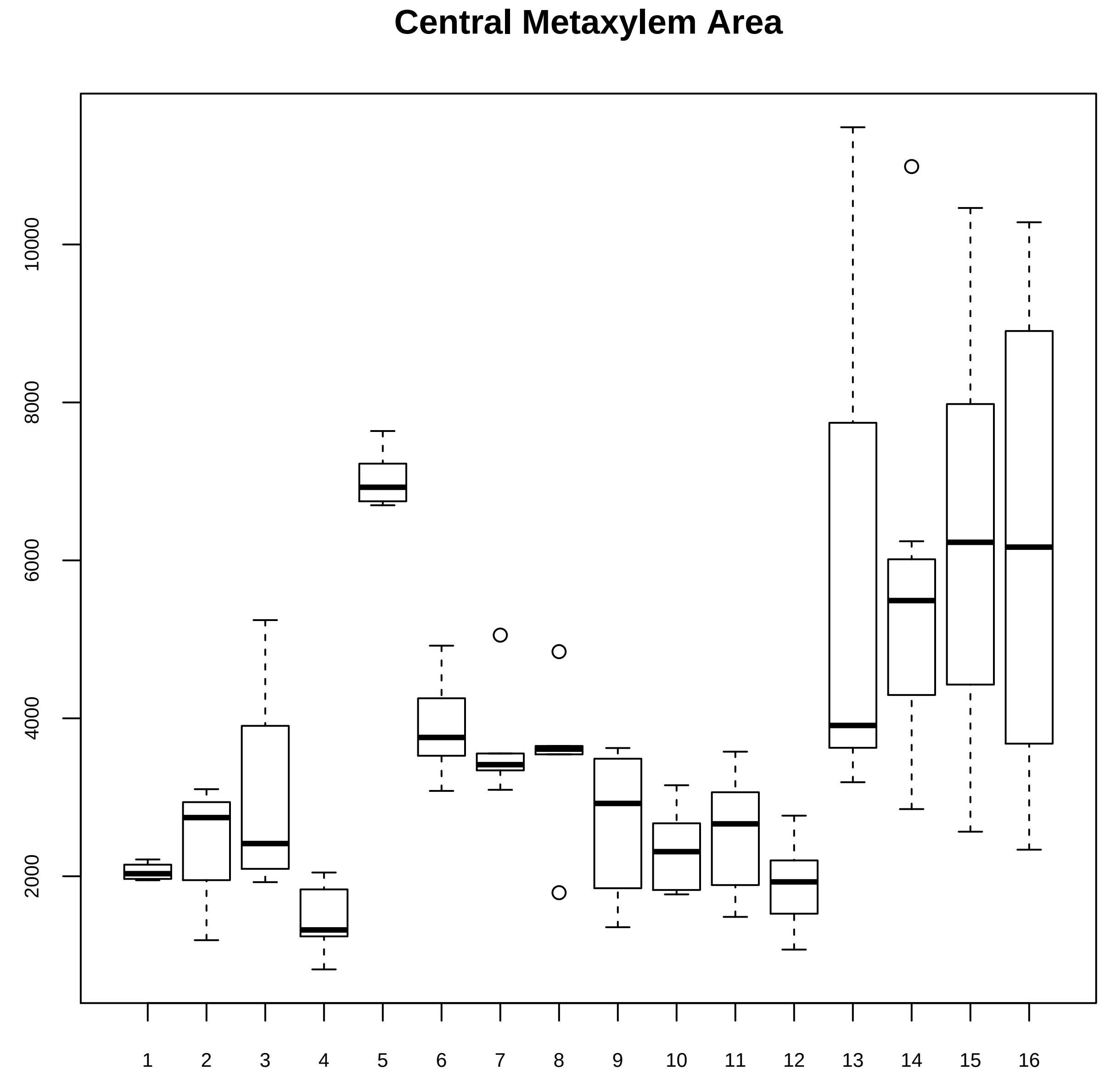

Central Metaxylem Area
10000
8000
6000
4000
2000
1
2
3
4
5
6
7
8
9
10
11
12
13
14
15
16

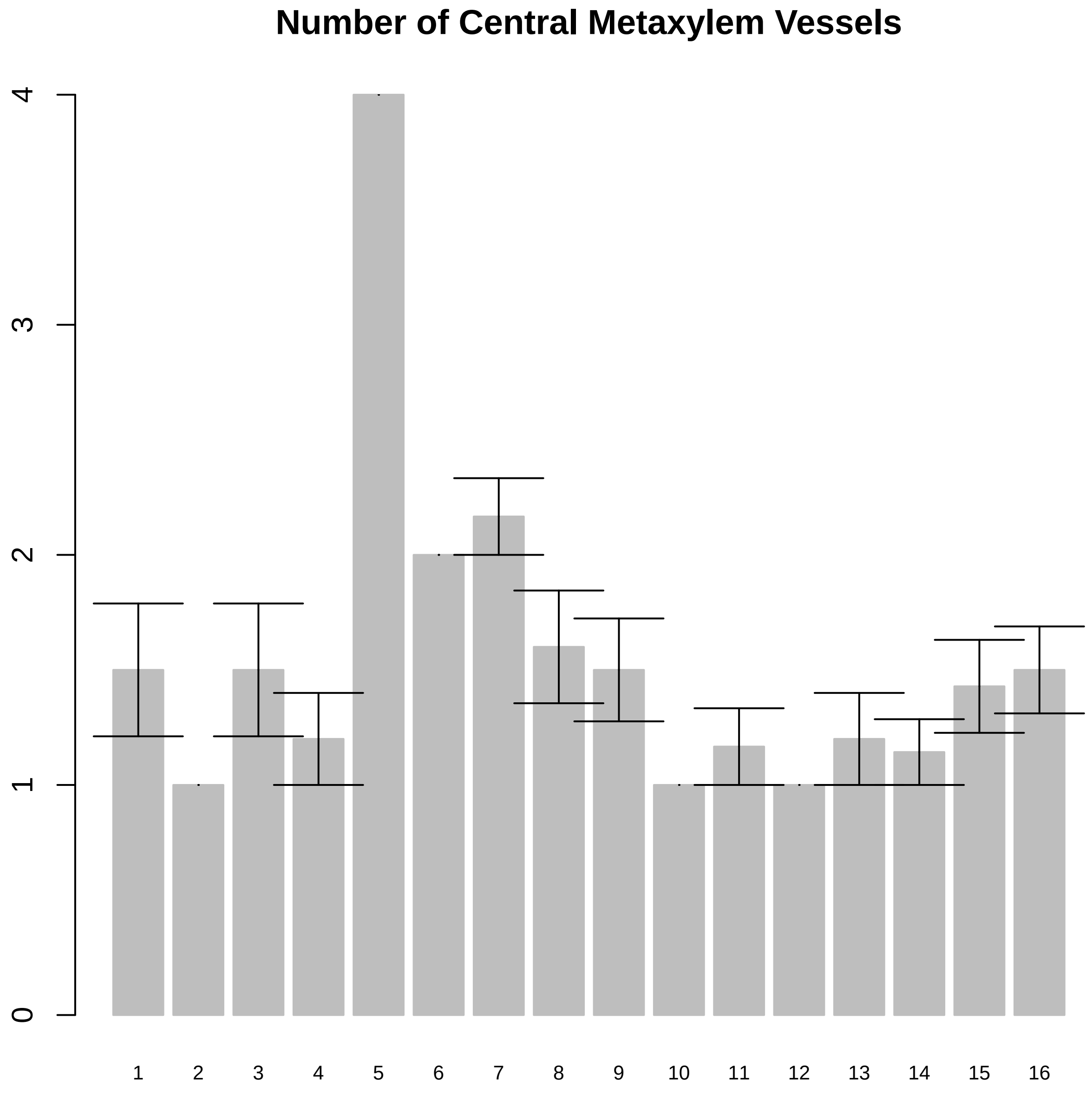

Number of Central Metaxylem Vessels
0
1
2
3
4
1
2
3
4
5
6
7
8
9
10
11
12
13
14
15
16

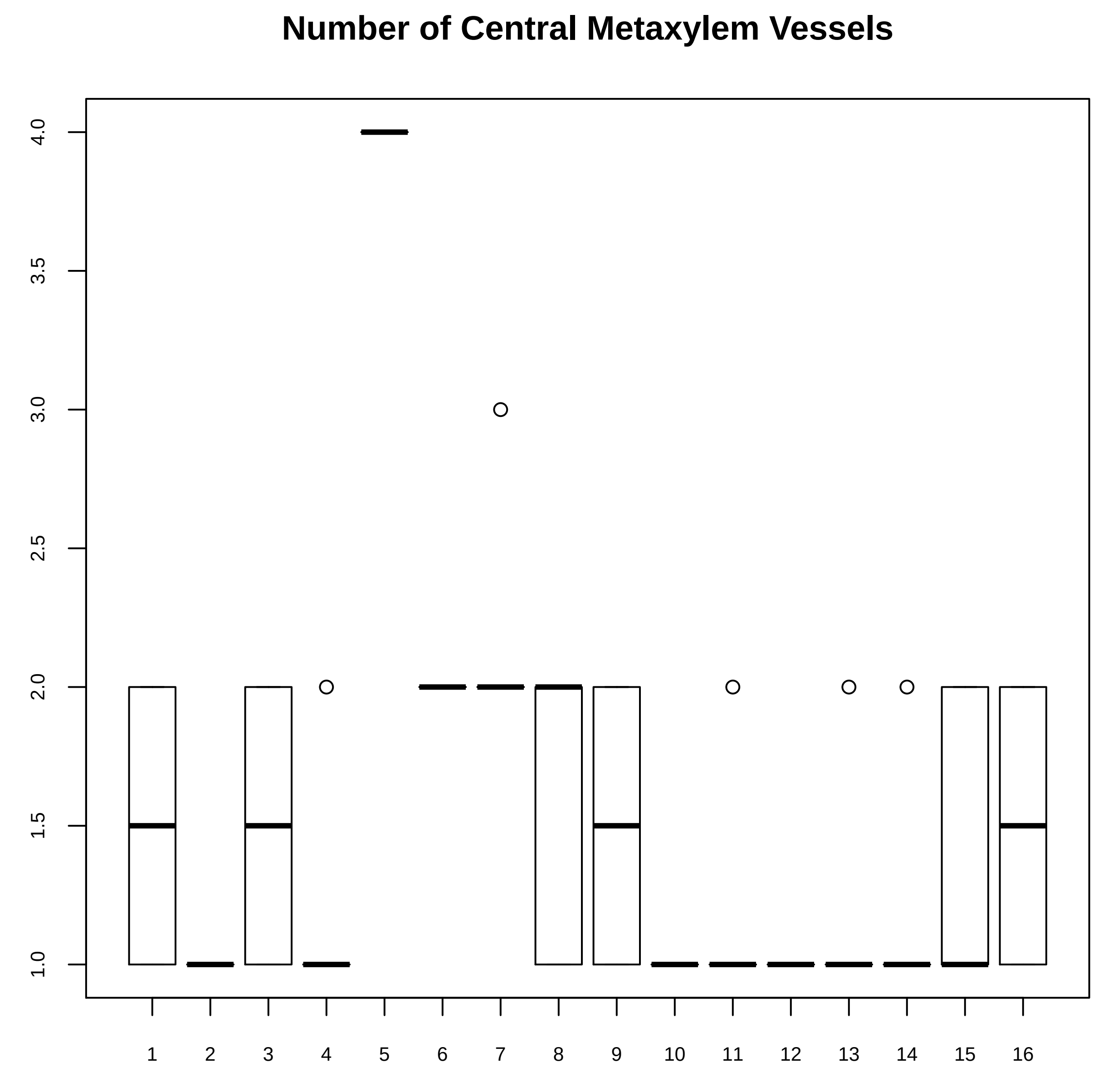
Number of Central Metaxylem Vessels
1.0
1.5
2.0
2.5
3.0
3.5
4.0
1
2
3
4
5
6
7
8
9
10
11
12
13
14
15
16

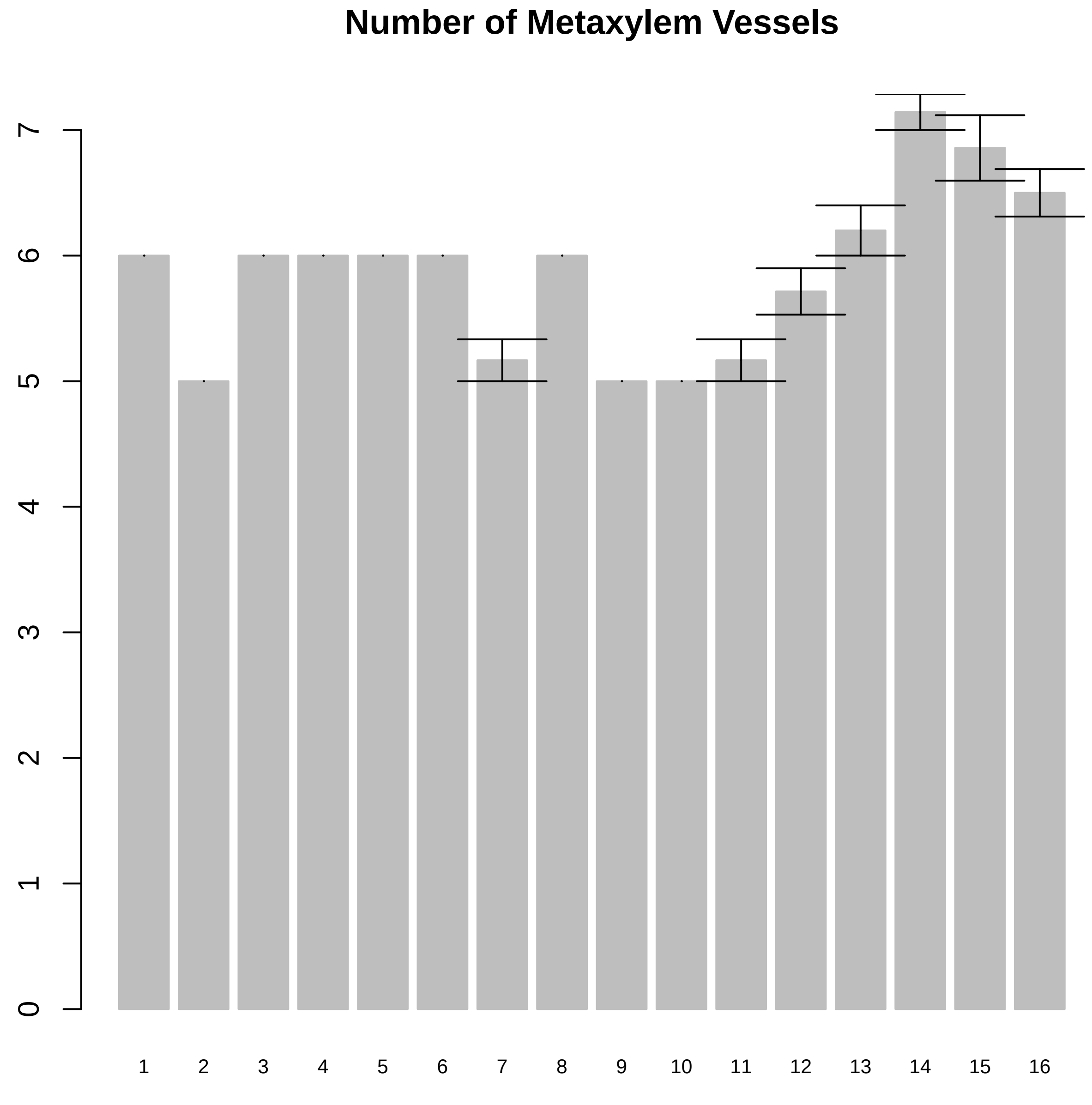

Number of Metaxylem Vessels
0
1
2
3
4
5
6
7
1
2
3
4
5
6
7
8
9
10
11
12
13
14
15
16

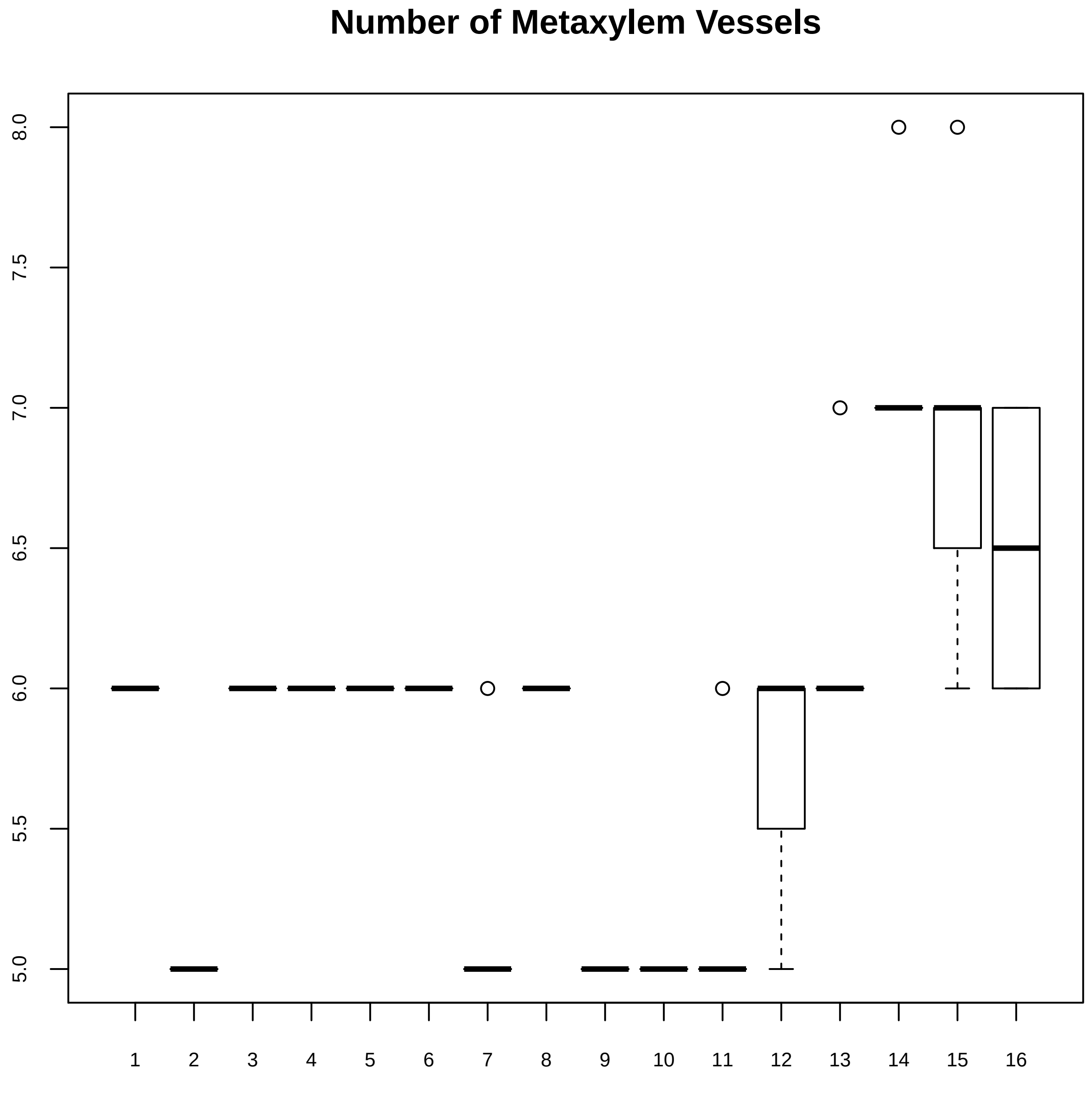
Number of Metaxylem Vessels
8.0
7.5
7.0
6.5
6.0
5.5
5.0
1
2
3
4
5
6
7
8
9
10
11
12
13
14
15
16

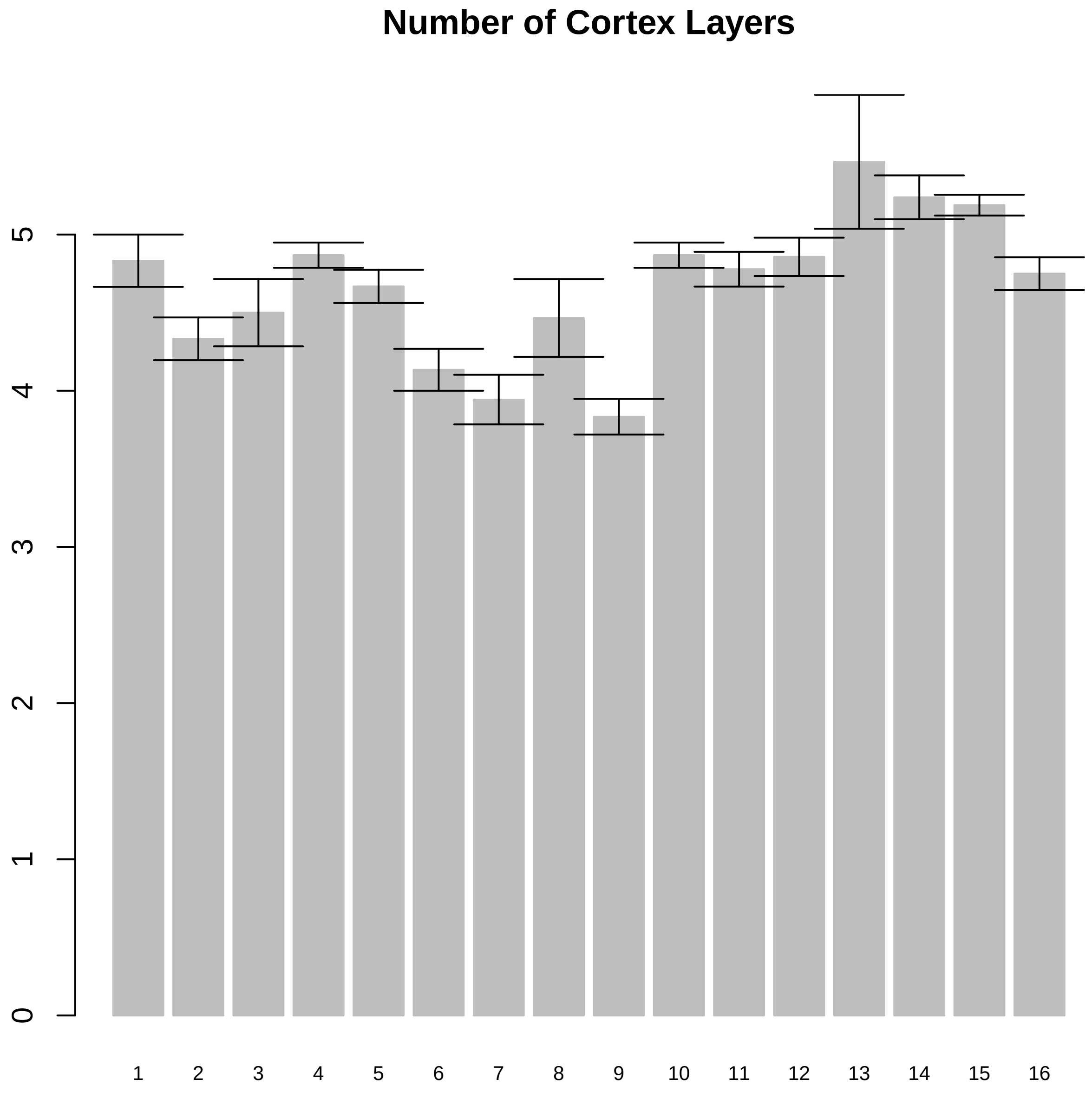
Number of Cortex Layers
0
1
2
3
4
5
1
2
3
4
5
6
7
8
9
10
11
12
13
14
15
16

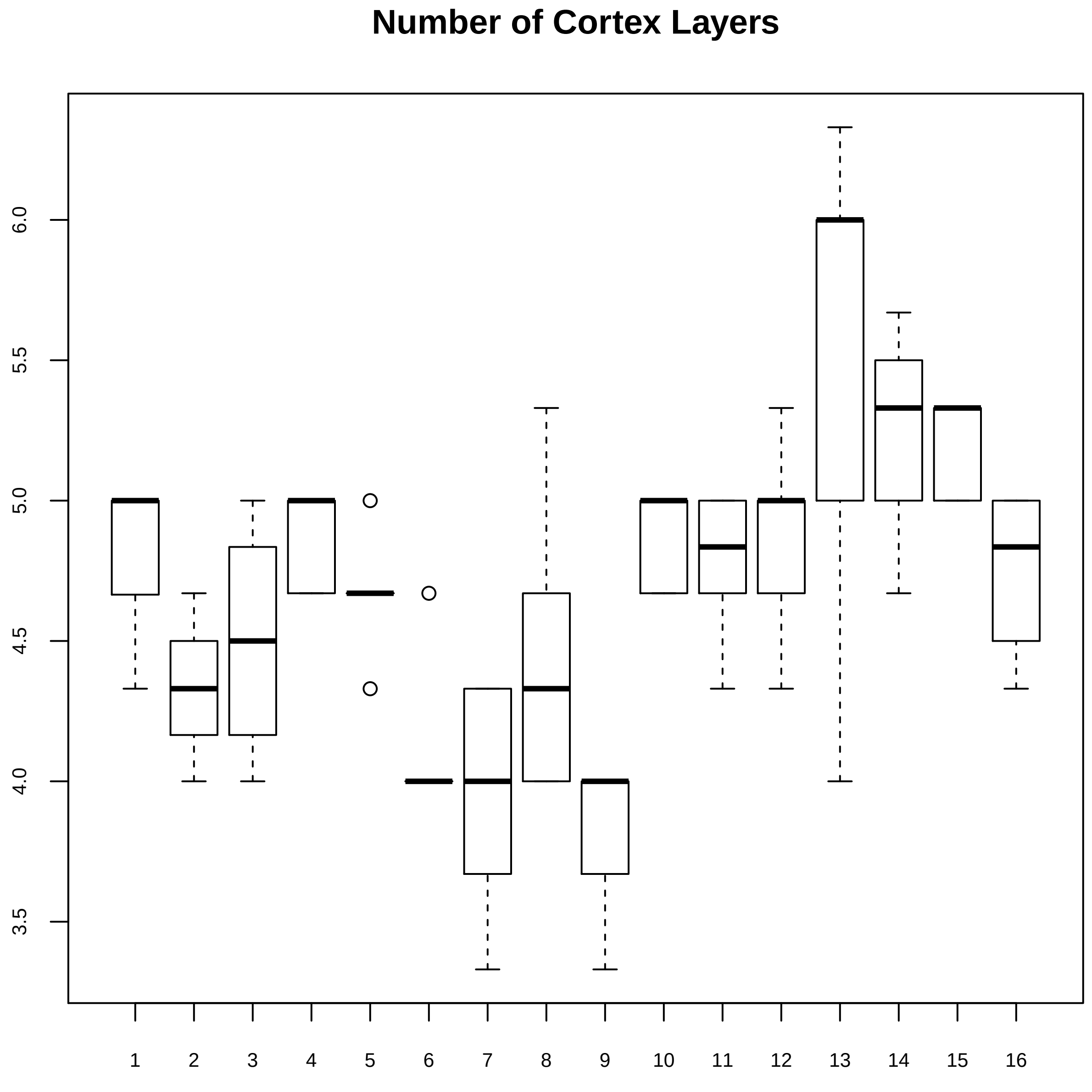
Number of Cortex Layers
3.5
4.0
4.5
5.0
5.5
6.0
1
2
3
4
5
6
7
8
9
10
11
12
13
14
15
16

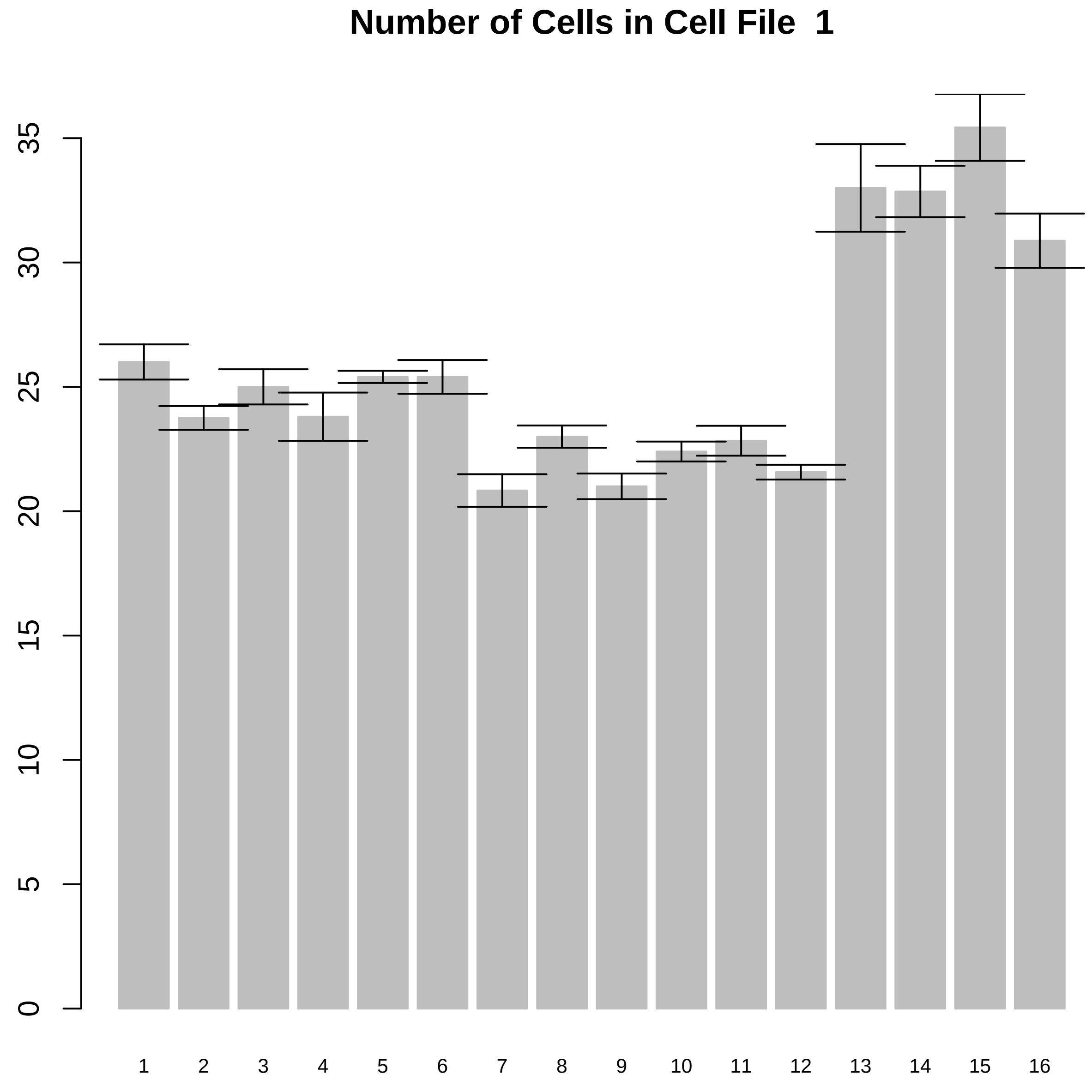
Number of Cells in Cell File 1
0
5
10
15
20
25
30
35
1
2
3
4
5
6
7
8
9
10
11
12
13
14
15
16

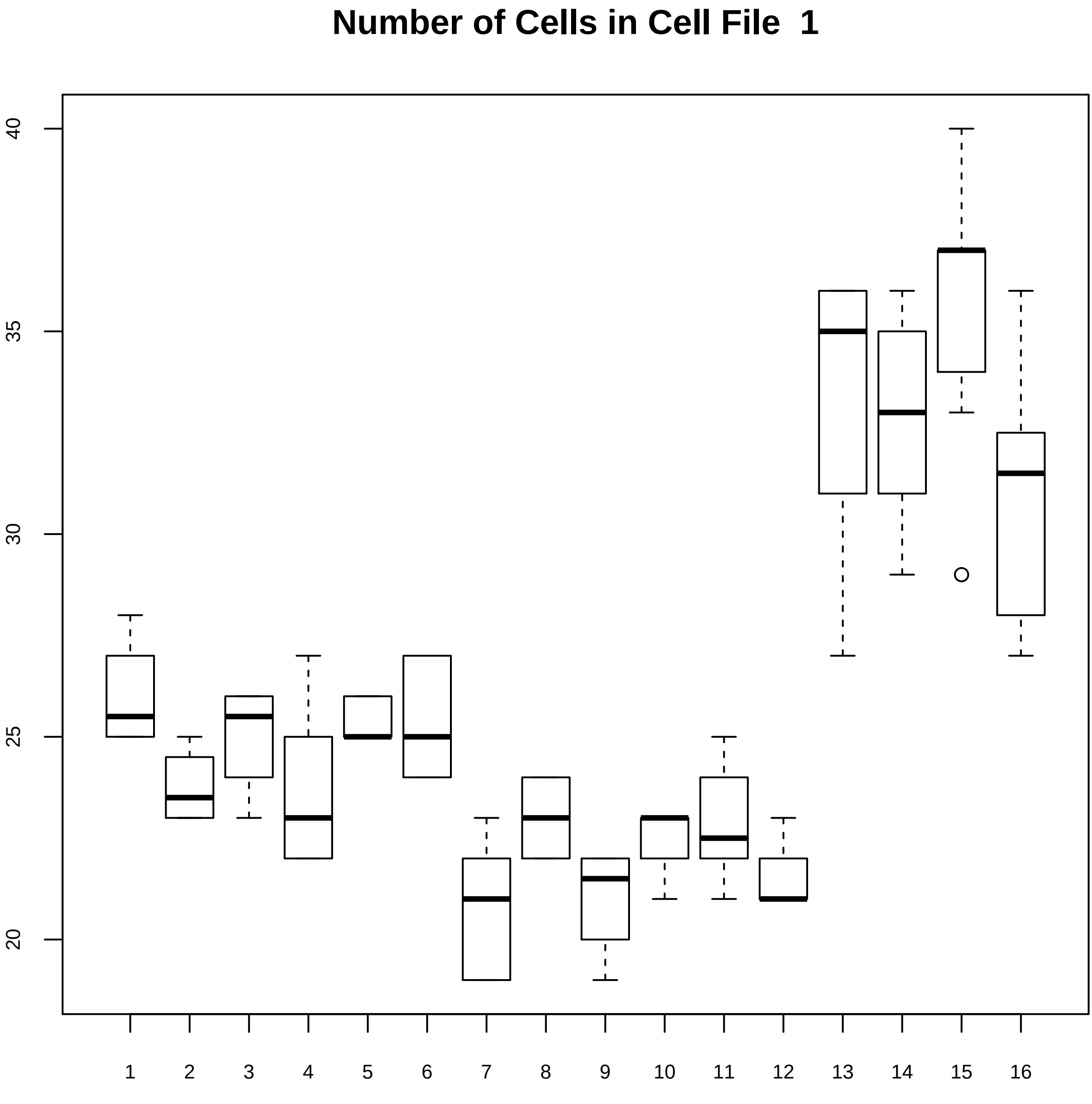

Number of Cells in Cell File 1
40
35
30
25
20
1
2
3
4
5
6
7
8
9
10
11
12
13
14
15
16

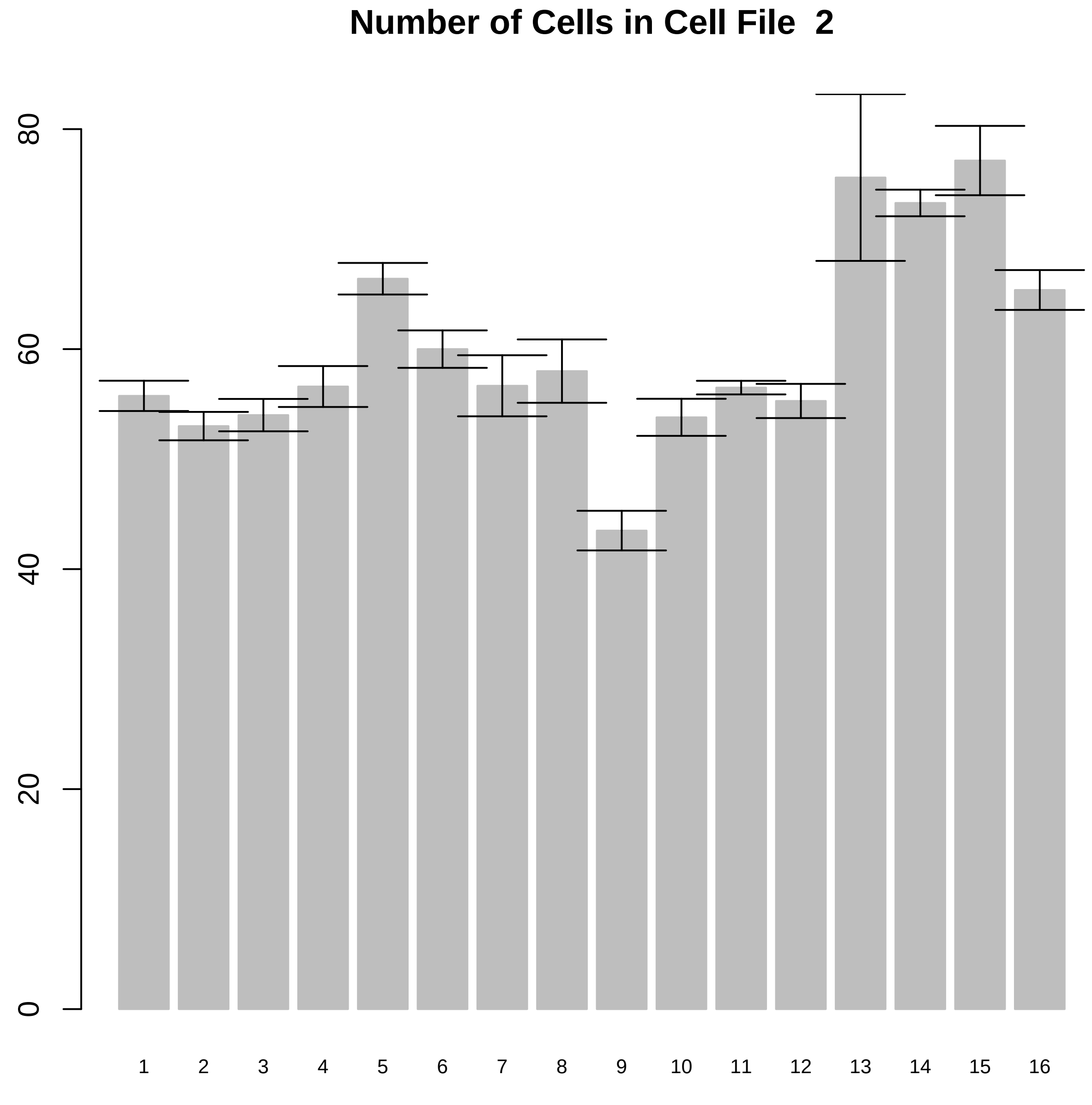
Number of Cells in Cell File 2
0
20
40
60
80
1
2
3
4
5
6
7
8
9
10
11
12
13
14
15
16

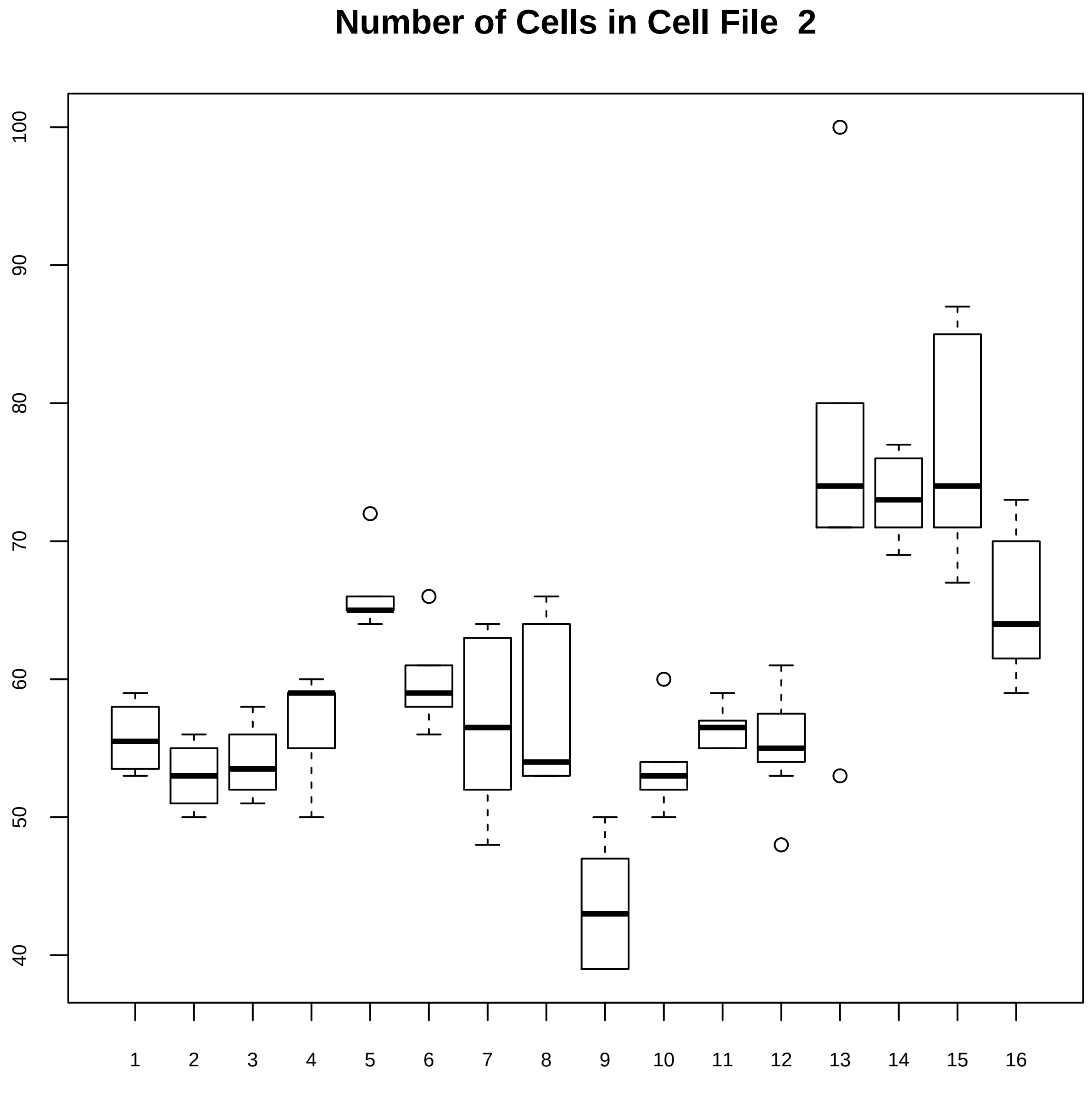
Number of Cells in Cell File 2
100
90
80
70
60
50
40
1
2
3
4
5
6
7
8
9
10
11
12
13
14
15
16
