# Supplementary material for: PHIV-RootCell: a supervised image analysis tool for rice root anatomical parameter quantification
Source: Front Plant Sci. 2015 Jan 19;5:790. doi: 10.3389/fpls.2014.00790 (PMC4298167; doi:10.3389/fpls.2014.00790)
Supplement: Supplementary file 4 [file Supplementary_Figure_Legends_and_Tables.DOC]

**Supplementary figure legends:**

**Supplementary Figure 1**. Means of 11 root parameters analyzed by 5 users (1 to 5). The coefficient of variation is indicated in parentheses for each feature. Areas are in pixels. For statistical significance of the data, a Newman-Keuls test was performed (letters a and b on the graphs).

**Supplementary Figure 2**. Means of 10 parameters analyzed on 16 rice varieties (1 to 16). Areas are in pixels. For statistical significance of the data, a Newman-Keuls test was performed (letters a,b,c,d,e on the graphs).

**Supplementary Figure 3**. Correlation circle of the PCA of 10 root anatomical parameters measured on 16 varieties of rice. The 2-dimensional representation preserves 86.7% of the variance between features. The first axis, PCA1, a vigor axis, is mainly determined by the areas and numbers of cells. The second axis, PCA2, is determined by the central metaxylem features (high positive correlation with the number of central metaxylem vessels and to a lesser extent with the central metaxylem area and negative correlation with the number of cortex layers).

**Supplementary Table 1. Example of an “Analyses” window.**

**Supplementary Table 2. Phenotypic correlations between 10 traits measured on 16 varieties.**
