# Supplementary material for: PHIV-RootCell: a supervised image analysis tool for rice root anatomical parameter quantification
Source: Front Plant Sci. 2015 Jan 19;5:790. doi: 10.3389/fpls.2014.00790 (PMC4298167; doi:10.3389/fpls.2014.00790)

Suppl. Figure 1

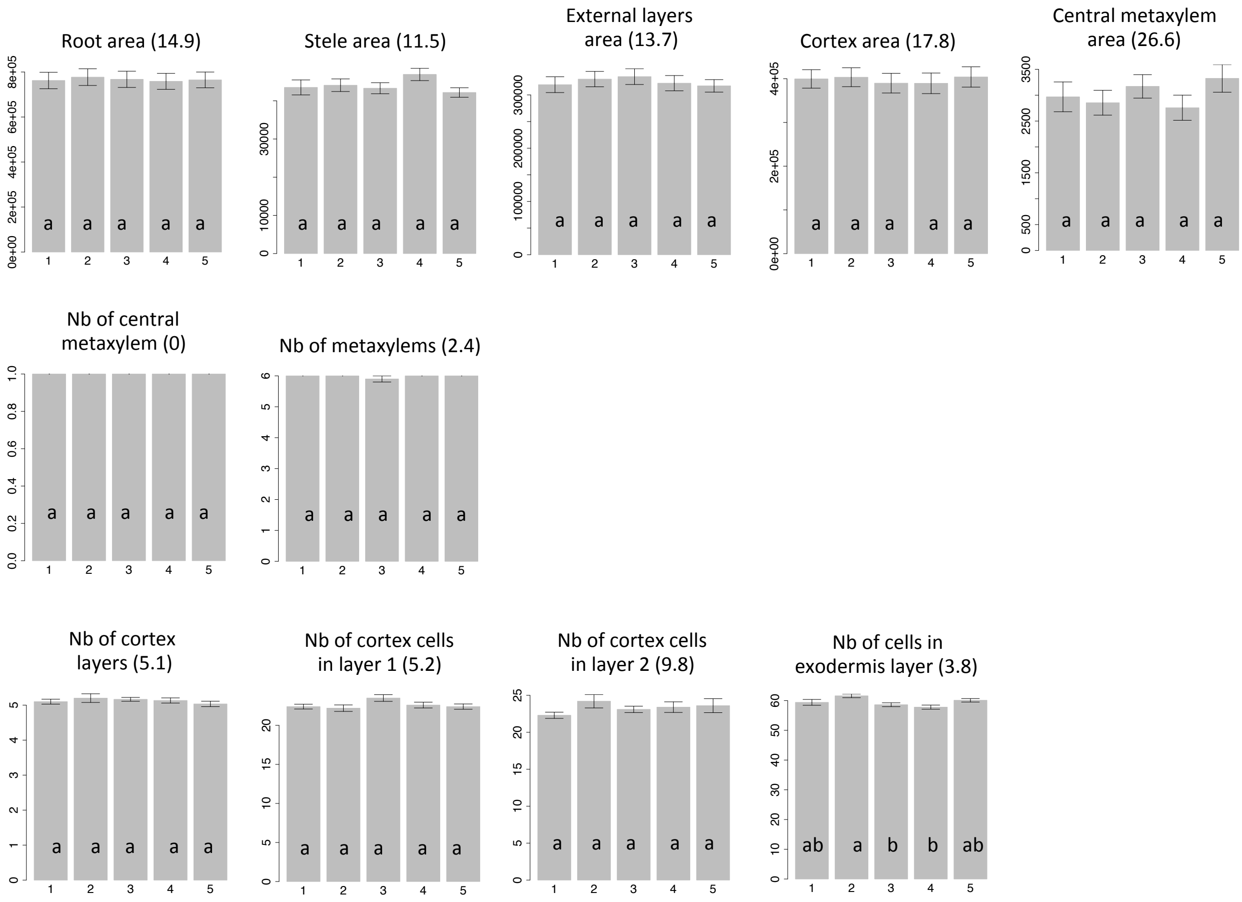

Suppl. Figure 2

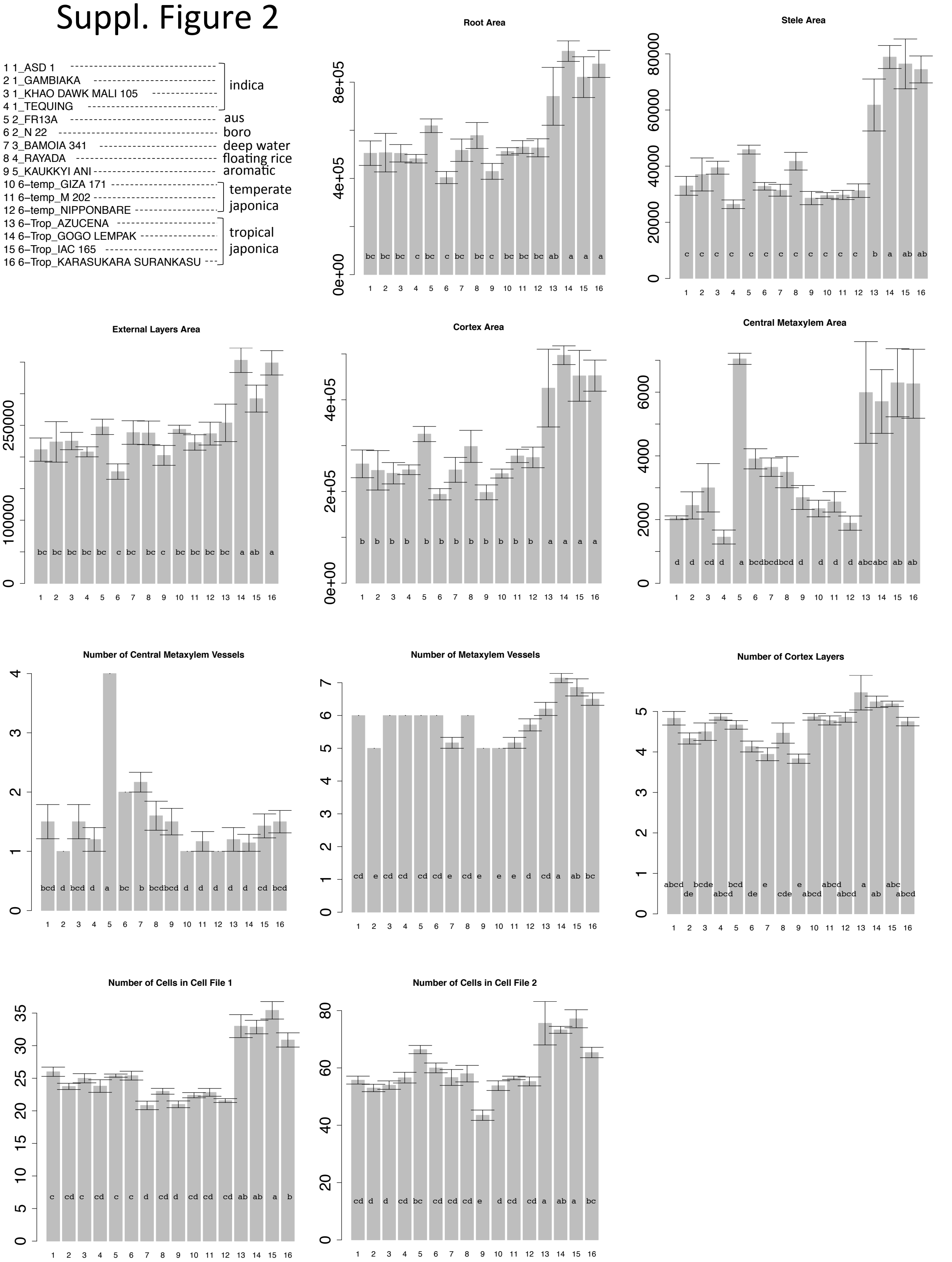

Suppl. Figure 3

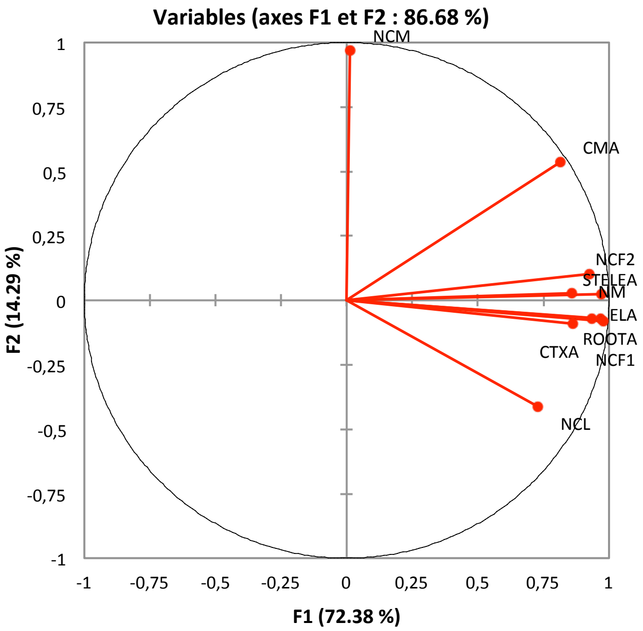

Supplement: Supplementary file 5 [file Supplementary_Figures.PDF]
