# Supplementary material for: PHIV-RootCell: a supervised image analysis tool for rice root anatomical parameter quantification
Source: Front Plant Sci. 2015 Jan 19;5:790. doi: 10.3389/fpls.2014.00790 (PMC4298167; doi:10.3389/fpls.2014.00790)
Supplement: Supplementary file 6 [file User-manual.PDF]

PHIV-RootCell toolset user manual

The PHIV-RootCell program is a macro toolset running on the 1.48 h (or higher) version of ImageJ (http://imagej.nih.gov/ij/download.html).

To run the PHIV-RootCell toolset for the first time, the "Polar Transformer" plugin is needed (http://rsbweb.nih.gov/ij/plugins/download/Polar_Transformer.class) and has to be saved in the plugins folder (ImageJ/plugins/).

The text file entitled "PHIV_Rootcell_toolset.txt" has to be saved in the ImageJ/macros/toolsets folder.

Once installed, you will be able to run the toolset:

1-In the tool bar menu, click on the "More Tools" menu (">>") and select « PHIV_Rootcell_toolset ».

2-Note that this user manual can be displayed by clicking on the " ? " button in the PHIV-RootCell tool bar. However, do not modify this file because it also contains the program.

3- Click on the « Parameters » button if you want to modify (i) the verbose mode (default: Yes), or (ii) the type of image you will analyze (default: Fluorescence) or (iii) the unit measure (default: Pixel).

4-From the ImageJ File/Open menu, select an image of a transverse root section to analyze.

5-In the tool bar menu, press the R button (Root Selection).

6-Adjust the threshold in the "Threshold" window to cover the whole root if necessary.

7-Select the area by clicking on it with the automatically selected "Wand (tracing) tool".

8-Press OK in the "Root Selection: step 1/5" window. You have the opportunity to correct the selection by using the automatically selected "brush tool" (size = 200). Click in the center of the root, and correct the selection by moving the circle around.

9-Press OK in the "Option" window. In the "ROI Manager" window, select "Show All" and "Labels". You will see on your image the selected area for the root, and in the center, the number "1" corresponding to the first selection. In the "ROI Manager" window, "Root" selection is also now displayed. From this window, you can highlight this selection and delete it or update it at any time if you want.

10-In the tool bar menu, press the S button (Stele Selection).

11-Adjust the oval selection to the stele perimeter.

12-Press OK in the "Stele Selection: step 2/5" window.

13-In the tool bar menu, press the X button (Xylem Selection and Count).

14-Adjust the threshold in the “Threshold” window to reveal central metaxylem vessel(s). If several vessels have to be selected (complex selection), press the shift key. In this case, the number of central metaxylem vessels (clicks) will be recorded. The default value for this number is 1.

15-Validate the number of central metaxylem vessels and press OK. The default value is 1.

16-Validate the number of metaxylem vessels and press OK. The default value is 6.

17-In the tool bar menu, press the C button (Cortex Selection).

18-Adjust the threshold in the “Threshold” window to specifically select inner tissues (delimited by sclerenchyma).

19-Select the area by clicking on it with the automatically selected "Wand (tracing) tool".

20-Press OK in the "Cortex Selection: step 4/5" window. You have the opportunity to correct the selection by using the automatically selected "brush tool" (size = 100). Click in the center of the root, and correct the selection by moving the circle around.

21-Press OK in the "Option" window.

22-In the tool bar menu, press the L button (Layer and Cell File Counts).

23-Depending on the number of measures (n) defined by user for the number of layers (in options, default = 3), draw a vertical line starting in the center of a cell in the first layer up to the center of a cell in the last layer to be counted and press OK in the "Number of cell layers" window.

24-Validate the number of cell layers counted.

25-Repeat this step n times.

26-Depending on the number of measures (n) defined by the user for the number of cell files (in options, default = 6), draw a horizontal line starting in the center of a cell in a cell file up to the center of a cell in the same cell file and press OK in the "Number of cells in cell file" window. Note that if the quality of your image is not good enough to draw the line on the entire window, you can do the measurement on a smaller portion, and the total number of cells will be extrapolated.

27-Validate the number of cells in the cell file counted.

28-Validate the extrapolation if only a portion of the image has been analyzed.

29-Repeat this step n times.

30-Press OK in the message window.

31-In the tool bar menu, press the D button (Data Display).

32-A pop-up window asks you the name of the dataset. If you want to analyze your data with the R script provided (PHIV_RootCell.R), you will have to fill it. For example, if you want to

compare two varieties (A and B), you will create 2 datasets (containing several picture analyses) named A and B.

33-Press OK in this window.

34-In the tool bar menu, press the N button (New image) to analyze a new picture.

35-At the end of your analysis, from the "Analyses" window, save the file: File/Save as... in text format (.txt).
